# Development of Two-Dimensional Classroom Discourse Analysis Tool (CDAT): scientific reasoning and dialog patterns in the secondary science classes

**DOI:** 10.1186/s40594-018-0100-0

**Published:** 2018-02-19

**Authors:** Soon C. Lee, Karen E. Irving

**Affiliations:** 1 Wichita State University, Wichita, KS USA; 2 The Ohio State University, Columbus, OH USA

**Keywords:** Classroom discourse, Discourse analysis, Scientific reasoning, Formative feedback, Scientific discourse

## Abstract

**Background:**

In a science classroom, students do not simply learn scientific ways of doing, knowing, and reasoning unless they find ways of appropriating scientific discourse. In the Next Generation Science Standards, major forms of *scientific discourse* are emphasized as a main part of the Science and Engineering Practices. To enhance student engagement in scientific discourse, teachers need to help students differentiate scientific ways of talking from *everyday ways of talking*. Thus, science teachers should be able to be aware of the differences to provide opportunities for students to engage in scientific discourse.

**Results:**

In this study, the *classroom discourse analysis tool* (CDAT) was developed to help science teachers and educators identify the patterns of their classroom discourse with the lens of scientific reasoning. The CDAT suggests a new way of discourse pattern finding with the two-dimensional graphic organizer and the quantitative data produced by the coding. To pilot the CDAT analysis, 13 videos and transcripts of two middle and one high school teachers’ physical science classes were viewed and analyzed. The results from CDAT coding show illustrative information that characterizes the classroom discourse patterns in relation to scientific reasoning and teachers’ questioning and feedback. A coded CDAT table shows what reasoning components used in the classroom dialogs between the teacher and students. It also shows how students engaged in the dialogs with the variations of their answers by the teacher’s question and feedback.

**Conclusion:**

The results show the patterns of students’ responses strongly depend on teacher’s question or feedback. In addition, this analysis also generates various quantitative data that represent certain characteristics of the classroom discourse, i.e., length of dialog and the number of reasoning components used. The possible implications of CDAT analysis are to explore the relationships between teachers’ discourse patterns and students’ achievement along with changes in their reasoning skills. Student attitudinal outcomes such as motivations, interests, or self-efficacy could also be compared by the classroom discourse patterns revealed by CDAT. CDAT analysis itself can also be used in a teacher professional development as an intervention to help teachers see their classroom discourse patterns.

**Electronic supplementary material:**

The online version of this article (10.1186/s40594-018-0100-0) contains supplementary material, which is available to authorized users.

## Background

Classroom discourse often refers to language-in-use that teachers and students employ to communicate with each other (Cazden 2001; Gee 2004b; Rymes 2015). In a science classroom, students do not simply learn scientific ways of doing, knowing, and reasoning unless they find ways of appropriating scientific discourse (Bromme et al. 2015; Gillies and Baffour 2017; Lemke 1998; Scott 2008). Scientific discourse has been used interchangeably with “talking science” or “discourse of science” that scientists use for their own sense-making purposes (Gee 2004a; Lemke 1998; Scott 2006). The importance of student engagement in scientific discourse has been emphasized in the National Science Education Standards, which recommend “[orchestrating] discourse among students about scientific ideas” (National Research Council 1996, p. 32). In the Next Generation Science Standards, scientific discourse is emphasized as main parts of the science and engineering practices (i.e., “Engagement in practices is language intensive and requires students to participate in classroom science discourse.” – Appendix F) (Lee et al., 2014a; National Research Council, 2012, 2013, 2015).

Studies have also shown that teachers need skills to facilitate scientific discourse not only to improve students’ inquiry and reasoning skills (Gillies and Baffour 2017; Hardy et al. 2010; Watters and Diezmann 2016) but also to enhance students’ engagement in productive science practices (Hardy et al. 2010; National Research Council 2012; Webb et al. 2006). Literature also suggests that teachers need to help students differentiate and transfer from everyday ways of talking to scientific ways of talking by engaging students in various forms of discourse as much as possible (Duschl and Osborne 2002; Gillies and Baffour 2017; National Research Council 2013; Nystrand and Gamoran 1991; Scott 2006; Viennot 1979). Therefore, science teachers should be able to be aware of the differences in their classroom discourse to provide opportunities for students to engage in scientific discourse (Gillies and Baffour 2017; Gunckel et al. 2009; Hardy et al. 2010; Windschitl et al. 2008).

In this study, the Classroom Discourse Analysis Tool (CDAT) was developed to help science teachers be aware of their classroom discourse patterns through the lens of scientific inquiry and reasoning. The CDAT coding produces illustrative information that shows visualized discourse patterns in relation to scientific reasoning, teachers’ questioning and feedback, and students’ engagement in the discourse. In this paper, the features of the CDAT are presented with a theoretical framework, development process, assessment methods, the reliability and validity of the CDAT, and what the analysis results show with examples from three high school physical science teachers’ classrooms.

## Theoretical framework

Gee (2004a) defined “a discourse” as a socially accepted association among ways of using language, of thinking, and of acting that can be used to identify oneself as a member of a socially meaningful group. Researchers have also claimed that a process of learning involves mastering the ways of the discourse in the community e.g., science, law, or arts. (Bromme et al. 2015; Cazden 2001; Lemke 1998; Scott 2006). In this way, the term scientific discourse has been used to represent a process and/or a way to talk about scientific information, ideas, or practices and often involves talk about scientific methods, reasoning, and vocabularies. Lemke (1998) used the term “talking science” interchangeably with scientific discourse that covers “scientific statement,” “scientific argument,” “scientific explanation,” or “scientific discussion” throughout his book, *Talking science: Language, learning, and values.* In this study, scientific discourse is defined as talking about scientific knowledge and processes associated with scientific inquiry and reasoning.

### Scientific discourse vs. everyday discourse

In science education, the relationship between scientific and everyday ways of talking has been examined for understanding how students learn science and how best to teach science (Bromme et al. 2015; Nystrand and Gamoran 1991; Scott 1998). Two contrasting ways of framing the relationship between scientific and everyday have been suggested with different implications for learning and teaching (Moje et al. 2004; Rosebery et al. 1992). The first view regards everyday ways of talking and knowing as discontinuous with those of science and as barriers to science learning (Warren et al. 2001). Research on misconceptions maintain that students’ proper understanding of the target concept are usually hindered by everyday ideas that need to be replaced with correct conceptions (Gee 2004a; Warren et al. 2001). Gee (2004b) argues that everyday language limits students’ access to the knowledge of the discipline and obscures the details of causal, or other systematic relations among variables in favor of rather general and vague relationships. In this view, therefore, scientific discourse practices may depend on avoiding everyday knowledge from a personal experience that is not an adequate warrant for a scientific claim (Gee 2004a, b).

The second view, on the other hand, considers the relationship as a variety of complex forms with similarity, difference, complementarity, and/or generalization. Thus, studies with this view have focused on understanding the productive conceptual, meta-representational, linguistic, experiential, and epistemological resources that students have for advancing their understanding of scientific ideas (Clement et al. 1989; Moje et al. 2004; Scott 1998). In this view, everyday ways of talking are not seen as barriers but rather are considered as “anchoring conceptions” or “bridging analogies” to assist students in developing their understanding of scientific ways of knowing (Clement et al. 1989; DiSessa et al. 1991). According to Lemke (1998), the conflicts between everyday and scientific ways of talking can also be an integral point of interest between teacher and students in the classroom through the activities they do.

### Scientific reasoning and everyday reasoning

The contemporary view of scientific reasoning encompasses the procedures of scientific inquiry including *hypothesis generation*, *experimental design*, and *evidence evaluation* and *drawing inferences* (Anderson 2006; Koslowski 1996; Kuhn and Franklin 2007; Kuhn and Pearsall 2000; Wilkening and Sodian 2005; Zimmerman 2007). Zimmerman (2007) categorized the three major cognitive components of scientific reasoning: searching for hypotheses, searching for data or evidence from experiments or investigations, and *evidence evaluation*. Kuhn and Franklin (2007) have also argued that the defining feature of scientific reasoning is the set of skills involved in differentiating and coordinating theory and evidence with interest in both the inductive processes involved in the generation of hypotheses and the deductive processes used in the testing of hypotheses (Kuhn and Franklin 2007; Zimmerman 2007). Both inductive and deductive reasoning processes involve the coordination of theory and evidence, which leads to enhanced scientific understanding (Koslowski 1996; Kuhn and Franklin 2007; Zimmerman 2007).

Anderson (2006) redefined everyday reasoning as how students make sense of the world by building understanding through *everyday pattern finding* and *everyday storytelling* compared to how scientists do *scientific reasoning*. In this study, *everyday reasoning* refers to an individual’s construction of intuitive theories about their experiences with natural phenomena, which may or may not match currently accepted scientific explanations of those same phenomena (Anderson 2006; Brown and Clement 1989; Perkins et al. 1983; Vosniadou and Brewer 1992). Students often bring these theories to the classroom as resources to help them as they think out loud (Rosebery et al. 1992; Warren et al. 2001). These theories have, typically, no explanatory power and cannot be qualified as scientific (Anderson 2006; Perkins et al. 1983; Warren et al. 2001). Several types of *everyday reasoning* as a process of sense making of the world have been identified i.e., *phenomenological primitives* (p-prims), *force-dynamic reasoning*, and *learning progressions* (DiSessa 1993; Gunckel et al. 2009; Talmy 1988). These classifications of students’ sense-making processes have provided insightful understanding of student’s reasoning compared to scientists’ reasoning in relation to cognitive models.

### Science classroom discourse analysis

Two types of research have been conducted to assess or analyze science classroom discourses. Ones have been mainly focusing on dialogs that happen in the classrooms, e.g., the *Critical Discourse Analysis* (CDA) by Gee et al., *Semantic and Thematic discourse analysis* by Lemke, and Dialogic Teaching-and-Learning by Rojas-Drummond et al. (Gee 2011; Hackling et al. 2010; Hicks 1996; Lemke 1998; Rojas-Drummond et al. 2013). These tools use a dialog between a teacher and students as the unit of episode and an utterance between them as the unit of coding (Gee 2011; Hackling et al. 2010; Lemke 1998). The others evaluated the activities along with discourse in a science classroom using Likert-type questionnaires, i.e., *Reformed Teaching Observation Protocol* (*RTOP)*, *Electronic Quality of Inquiry Protocol (EQUIP)* (Brandon et al. 2008; Piburn et al. 2000; Weiss et al. 2003). The observation protocols use various evaluation criteria to judge the classroom discourse and activities that includes teacher interview and questions for detailed descriptions of the classroom characteristics (Brandon et al. 2008; Piburn et al. 2000; Weiss et al. 2003).

However, several issues with Likert-type observation protocols have been revealed and discussed. First, these protocols require coders to make holistic judgments about broad categories of lesson and instruction and to rate each item for the class period. The abstract quality of the Likert scale makes the protocols more difficult to use by teachers or practitioners in a formative sense without extensive training, i.e., what does it mean by being rated as 2 or 4 for a given indicator? Although most of them provide an operational definition or concept, some studies have still found a high level of variation among the coders within the same study (Amrein-Beardsley and Popp 2012). Second, the protocols were designed to evaluate specific types of instructions (i.e., inquiry-based) or to be used by particular groups of people (i.e., researchers). Thus, these instruments may result in poor and invalid results by not being used for the purpose for which they were intended (i.e., to evaluate other types of science instructions or used by teachers or instructional coaches). The greatest problem for the Likert-based instruments is multi-collinearity in that there is a substantial correlation repeatedly seen between all the individual items and the overall lesson score. Even though each item has a unique aspect to evaluate the instruction, significant overlaps exist in the data measured by the indicators. Thus, it is often hard to distinguish clearly between different levels of performance on the overlapped aspects (Lund et al. 2015; Marshall et al. 2011; Sawada et al. 2002). Lastly, these observational protocols were not developed to examine teachers or students’ level of reasoning skills used in their classroom discourses.

Only a few studies have examined students’ conceptual change through classroom discourse because of the practical challenges of measuring the level of reasoning and understanding separately (Duschl and Gitomer 1997; Hardy et al. 2010; Nystrand and Gamoran 1991; Nystrand et al. 2003). Hardy et al. (2010) argued that conceptual understanding and level of reasoning are intertwined in classroom discourse, and they effectively analyzed these two dimensions separately with four reasoning levels of claim justification and three levels of conceptual understanding. Nystrand and Gamoran (1991) analyzed the classroom discourse by coding authenticity, uptake, level of evaluation of the teachers’ questions, and responses. The study of Duschl and Gitomer (1997) identified three levels of assessment conversation in science classrooms: (1) the first stage is to receive student ideas, (2) the second stage is to recognize information from the students, and (3) the third stage is to use the information. According to Nystrand and Gamoran (1991), whole-class discourse in secondary school could be dominated by claims that are unsupported by empirical evidence (Nystrand and Gamoran 1991). In the secondary science classrooms, Hardy et al. (2010) also showed that the majority of the reasoning units scored on the lowest level for the claim, that is, no evidence was produced to support or refute a claim (Hardy et al. 2010). According to Nystrand and Gamoran (1991), significant academic achievement is not possible without sustained and substantive engagement guided by teachers in classroom discourse (Nystrand and Gamoran 1991). The studies also showed that classroom discourse with teachers’ assessment and feedback holding students’ substantive engagement could be a way of improving students’ scientific reasoning skills and conceptual understanding (Duschl and Gitomer. 1997; Hardy et al. 2010; Nystrand and Gamoran 1991). Although teachers’ questions, prompts, and feedback have significant effects on students conceptual understanding and reasoning skills (Duschl and Gitomer 1997; Hardy et al. 2010; Nystrand and Gamoran 1991), characteristics of teachers’ discourse and how they affect classroom discourse to be scientific need to be studied more.

The conceptual framework for the CDAT was constructed on the basis of the components and schemes of scientific reasoning with constructivist’s perspective, which highlights teaching and learning is a process of interactions with others (Amsterlaw 2006; Dolan and Grady 2010; Gillies and Baffour 2017; Howe and Abedin 2013; Piekny and Maehler 2013; Vygotsky 1978). The authors propose a system of two-dimensional analysis to investigate dialogs between a teacher and students in the context of science, consisting of “reasoning components” (RC) in one dimension; these are located in the columns of the CDAT coding table; “utterance types” (UT) in the other dimension, which are located in the rows. Although the coding scheme mainly focuses on the micro level, utterances, the results in a CDAT table illustrate macrolevel of classroom discourse between the teacher and students.

### Conceptual framework for bridging scientific discourse and everyday discourse

**Fig. 1:**
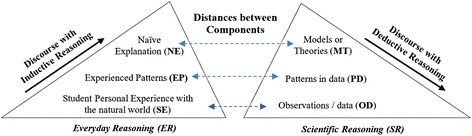
During a series of dialogs, a teacher can help students transfer their reasoning from everyday reasoning to scientific reasoning. The distances between the components in each ER and SR illustrate the relative difficulty of transformations. Thus, the most difficult transformation would be from NE to MT and the best transformation could happen from SE to OD followed by EP to OD and NE to OD (adapted from Anderson’ book, Teaching Science for Motivation and Understanding, 2006)

The conceptual framework (Fig. 1) is designed to illustrate the structures and major components of everyday reasoning and scientific reasoning and show possible pathways to transfer from the former to the latter. From the discussion about scientific reasoning above, *Data*, *Patterns from the data*, and *Theories* are selected as major components of scientific reasoning (Anderson 2006; Koslowski 1996; Kuhn and Franklin 2007; Zimmerman 2007). The process of scientific reasoning involves the coordination of theory and data which requires searching for or applying the patterns between them. In general, inductive reasoning starts with observations/data and moves to a model/theory generating new scientific knowledge, while deductive reasoning starts with a model/theory and moves to observations/data to describe or predict the natural world (Anderson 2006; Kuhn and Pearsall 2000; Zimmerman 2007). Likewise, in everyday life, we use the inductive and deductive reasoning in the process of everyday sense-making which involves continuous interplay of both reasoning styles. Thus, in a similar way, *Personal Experiences*, *Experienced Patterns*, and *Naïve Explanations* are considered as the main components of everyday reasoning (Anderson 2006; Lemke 1998). In the process of everyday reasoning, students often construct explanations about their experiences using authority figures (i.e., teachers, parents) or their intuitive beliefs from their experienced patterns (i.e., trial runs, repetitive observations). Therefore, the learning progression in student’s discourse or reasoning can be indicated by his/her awareness of the difference between the two types of reasoning components.

Because a person’s discourse is the window through his/her thoughts flow, the components of scientific and everyday reasoning in the conceptual model (Fig. 1) are also considered as the discourse components. Scientific discourse commonly includes (1) *data* associated with observing, questioning, collecting, and describing; (2) *patterns* with hypothesizing, designing, organizing, analyzing, comparing, and classifying; and (3) *theories* with evaluating, concluding, discussing, and generalizing (Duschl and Osborne 2002; Lemke 1998; Nystrand and Gamoran 1991; Scott 2008; Zimmerman 2007). In contrast, everyday discourse often involves a person’s experiences, his/her experienced patterns, and his/her own explanations. As an example of everyday discourse, a student might argue that “*heavier objects fall faster*” (experienced patterns) like “a rock hits the ground first and then a feather” (student experience), and that is “because heavier objects are more attracted to the Earth” (naïve explanation). While, as an example of scientific discourse, a science teacher might explain that (1) *if there is no air resistance*, a rock and a feather hit the ground at the same time (scientific observation/data), and (2) *data show* every object falls at the same rate regardless of their mass (patterns in data) because (3) the object’s acceleration is *directly proportional to* the gravitational force but is *inversely proportional to* the mass (scientific model).

In the model, the distances between the components of everyday reasoning and that of scientific reasoning represent the difficulty to transform into scientific ones. For example, if a teacher tries to change students’ naïve explanation (NE) directly into the scientific theory/model (MT), he/she would meet with little success. However, if a dialog starts with personal experiences (PE), which is closest to observations/data (OD), teachers would be more effectively able to assist students to have an idea of scientific data. In a similar way, when a student shares experienced patterns or naïve explanations, directing him/her to the scientific observations would be the better way for him/her to discover the inconsistencies of his/her reasoning. Thus, this model implies how teachers can better facilitate students’ scientific discourse through the closer pathways from everyday reasoning to scientific reasoning components. For instance, without a scientific observation, it is not possible for students to notice scientific patterns from it. Thus, from the model, it is suggested that assisting students to do a scientific observation first, and then they might be able to see the patterns better. This model is also well consistent with Moje et al.’s (2001) suggestions of four characteristics of classroom interaction to convert student everyday knowledge and experience into scientific ones (Moje et al. 2001).

## Objective and research questions

The objective of this study was to develop a science Classroom Discourse Analysis Tool (CDAT) with the lens of scientific reasoning. This study also set out to demonstrate how the CDAT analysis characterizes science classroom discourse about teachers’ questioning and feedback. The following research questions were explored:

1. Is CDAT coding an effective method for recording and analyzing the classroom discourse regarding scientific reasoning?
2. Is CDAT coding an effective method for recording and analyzing the classroom discourse regarding teachers’ questioning and feedback?

## Development of CDAT

### Reframing components of a science class

To pilot the science CDAT, 13 videos and transcripts of two middle and one high school teachers’ physical science classes were viewed and analyzed repeatedly. Then, major common components across the observed science classes were categorized (Table 1). The components of a science class were classified into two categories of *classroom activities* in which students do hands-on activities, group discussions, or presentations and *classroom dialogs* in which the teacher has conversations with the students. A classroom dialog typically consists of sequences of students’ and a teacher’s *utterances* (see Table 1). A teacher or students’ utterance that involves a reasoning component such as personal experiences or observations related to the topic of the lesson is considered as the unit of coding (Boyatzis 1998; Gee 2004a). In this study, a *dialog* is considered as a *unit of episode* that consists of a teacher and his/her students’ consecutive utterances as they keep talking about the same topic (Boyatzis 1998).

**Table 1:** Class activities vs. class dialogs between a teacher and students

Class activities	Class dialogs (between a teacher and students)			
	Dialogs off topic	Dialogs on topic		
Lab, hands-on activity, group work, discussion, demonstration	Managing or disciplining students’ behaviors Explaining about activities, grades, test, or etc.	Dialog 1	Dialog 2	
		Teacher’s utterances and students’ utterances	Teacher’s utterances and students’ utterances	

### A coding example in a CDAT table

**Fig. 2:**
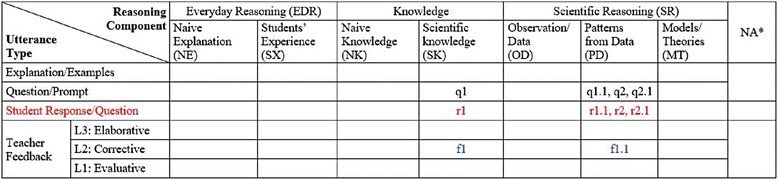
An example of CDAT table coded with everyday reasoning, knowledge, and scientific reasoning components

In the CDAT coding, each utterance is coded by two categories of *utterance type* and *reasoning component* arranged in a CDAT coding table (see Fig. 2). Utterance types such as teacher question (Q), teacher feedback (F), student response (R), or student question (SQ) are positioned in the rows in a CDAT table. While the reasoning components, i.e., naive explanation (NE), student experience (SX), scientific knowledge (SK), and observation/data (OD) are positioned in the columns in a CDAT table. The CDAT coding process includes two steps: first to determine the utterance type and second to determine the reasoning component of the utterance. Consequently, the code for an utterance is recorded in a cell where its reasoning component column meets its utterance type row (Fig. 2). If the teacher or students’ utterance is not relevant to the topic, the first question, it is coded in a cell of the last column, NA, in the table.

A CDAT table is typically used for coding two or three dialogs depending on the length of the dialogs. In a classroom, a dialog between a teacher and his/her students typically starts with a question (e.g., q1 in the example dialog no. 1) and ends with the teacher’s evaluative or corrective feedback with the answer disclosed (e.g., f1.1), and then, another dialog begins with a new question (e.g., q2). In this CDAT coding, individual dialogs are differentiated by numbers and the utterances in a dialog is coded with a decimal notation, i.e., 1.1, 1.2, or 1.3, until the dialog ends with a teacher feedback. As shown in example dialog no. 1, a new dialog is determined by a new question that requires a different answer than that in the previous dialog (i.e., q1, *What does that show?* and q2, *What happened here?*). Then, follow-up questions are often asked to confirm or help students understand or prompt other students’ responses (q1.2 and q2.2).

Example dialog no. 1

T: What does that show? (**q1 –** *coded in the cell of Scientific knowledge and Question/Prompt*)

SS: The slope. (**r1 –** *coded in the cell of Scientific knowledge and Student Response/Question)*

T: That’s the slope. (**f1.1–** *coded in the cell of Scientific knowledge and Feedback – L2)*

Was she going very fast? (**q1.1–** *coded in the cell of Patterns from Data and Question/Prompt)*

SS: No. (**r1.1 –** *coded in the cell of Patterns from Data and Student Response/Question)*

T: No, nice average speed. **(f1.1–** *coded in the cell of Patterns from Data and Feedback – L2)*

Example dialog no. 2

T: What happened here? **(q2 –** second dialogue starts here; *coded in the cell of Scientific knowledge and Question/Prompt*)

SS: She stopped. **r2**

T: Why does the line go straight? **q2.1**

SS: Time keeps going. **r2.1**

## Coding an utterance by reasoning components

For CDAT coding, definitions of each reasoning component were refined through the discussions among the authors in this study. First, the utterances about scientific concepts, laws, theories, principles, equations, or formulas are classified as scientific knowledge (SK). It is differentiated from the models and theories derived from the data or observations by students when communicated to assess students’ current knowledge. Second, observation/data (OD) is considered as descriptions of natural phenomena or quantified values collected from the hands-on activities or experiments in the classroom or from students’ experiences. However, utterances coded as OD do not include those resulting from inferential cognitive activities such as inductive or deductive reasoning, which belong to “patterns from data (PD).” Third, the utterances coded as PD typically involve certain types of reasoning such as comparison, contrast, relationship, diagram, graph, table, computation, categorization, and differentiation. Fourth, the utterances coded as models/theories (MT) only include a derived conclusion and an explanation from the students’ data analysis and pattern findings. However, without any data or evidence from students’ activities, utterances about a model or theory are coded as scientific knowledge (SK). Fifth, the utterances coded as student experience (SX) are not only limited to students’ personal experiences but also include second-hand experiences such as events or stories from a movie, history, or teacher’s experience. Sixth, the utterances coded as naive explanation (NE) include students’ theories based on everyday sense-making such as experienced patterns, a belief by authorities, or intuitive belief (Anderson 2006). Lastly, naive knowledge (NK) is considered as a form of common belief, legitimated by commonsensical opinions that are produced within home, work, and community interactions (Gardiner 2006; Lemke 1998; Moje et al. 2001).

As examples of utterances to be coded as NK or NE, a student might say “heavier objects fall faster” from his/her experiences or commonsensical opinion. Then, they might have an intuitive belief of “heavier objects are more attracted to the Earth” (naïve explanation) constructed by or through interactions with somebody in their community, i.e., parents, siblings, friends, or teachers.

### Coding the level of teacher feedback

The definitions and indicators for each teacher feedback level were drawn through reviews of literature on formative feedback, level 1––evaluative, level 2––corrective, and level 3––elaborative feedback (Hattie and Timperley 2007; Kluger and DeNisi 1998; Shute 2008). Table 2 shows the basic criteria of classification that are indicators for each level of feedback that includes types of content, delivery methods, and effect on student learning.

**Table 2:** Feedback levels

Level	Main aspects	Focus	Feedback content type	Delivery method	Effects on learning
1	Evaluative, normative	Student	Grade, praise, evaluation, comparison with others	General comments, no reason, attention to “self”, too long, vague, difficult, or interruptive students’ prompts	Negative or no effects
2	Corrective, verification	Task	Correction, right answer, direct hint, try again,	Short, clear, fast in written and spoken	Sometimes effective
3	Elaborative, facilitative	Task	Location of mistakes, addressing information, hint/cue for the direction, specific error (what and why)	No correct answers, manageable units for students, considering students’ level, specific and clear, goal orientation	Effective almost always

Synthesized from the studies of Hattie and Timperley 2007; Kluger and DeNisi 1998; Shute 2008

#### Level 1 feedback

According to the meta-analyses by Shute (2008), Kluger and DeNisi (1998), and Hattie and Timperley (2007), feedback that provides information too general or vague often hinders student learning. These types of feedback are considered as level 1 in CDAT coding. For example, evaluative feedback with a praise or subtle judgment is ranked as level 1. Level 1 feedback typically has vague information about correctness by just repeating students’ answers or simply saying “good” or “nice” in response. An example of level 1 feedback is shown in the dialog below, f1.2.

T: Well it is related to work. **f1** Work and? **qF1.1**

S: Amps! **r1.1**

T: It starts with an “E.” **f1.1**

S: Electricity! **r1.2**

T: Good! **f1.2**

#### Level 2 feedback

The key characteristic of level 2 feedback is providing a clear piece of information about whether an answer is correct or not; the most common way involves simply stating “correct” or “incorrect.” Teachers often use a question as this type of feedback, named *question Feedback* in this study. Two types of question Feedback (qF) were identified: one is confirming questions to make students’ answers explicit as level 2 feedback (qF7.6 in the dialog below); the other is elaborative questions to deepen students’ thinking as level 3 feedback, qF7.7 in the example dialog below.

S: Go down. **r7.5**

T: Go down? **qF7.6**

S: Stay the same. **r7.6**

T: It’s going to stay the same because what am I no longer doing? **qF7.7**

S: You’re not moving. **r7.7**

#### Level 3 feedback

Is elaborative responding to students’ answers and questions to guide students by addressing a specific error or providing examples. The example dialog below shows a level 3 feedback (qF7.7 above and F8.7 below*)* that provides a reason as well as a direction for further thinking rather than judging the student’s answer as right or wrong.

S: Flat. **r8.7**

T: The line’s going to be flat because I’m not gaining any more distance, the time is still passing. **f8.7**

### LOD

**Fig. 3:**
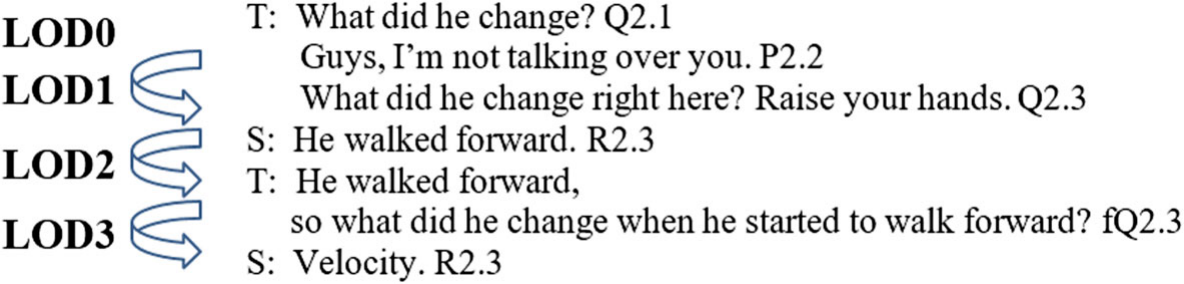
Length of dialog that counts the number of turn-changes between a teacher and students in the dialog

*Length of dialog* (LOD) represents a dialog pattern that counts the number of *turn-changes* between a teacher and students in the dialog (see Fig. 3). The number of utterances in a dialog does not necessarily agree with the LOD. As shown in Fig. 3, each LOD number indicates a specific discourse pattern. For example, a dialog of *LOD 0* indicates no student response yet, *LOD 1* indicates the first student response and no follow-up teacher feedback yet, *LOD 2* indicates a teacher feedback to the first student response. If the dialog ends here, it is considered as a typical question, response, and feedback (QRF) pattern; *LOD 3* indicates that this dialog includes two student turns in responses with one teacher feedback; *LOD 4* includes two turns in students’ responses and two turns in teacher feedback, etc. The longer LOD does not necessary imply that the dialogs are more productive or scientific. However, it can indicate that the dialog is beyond typical QRF discourse patterns and has more than two turns in student responses.

## Results from sample CDAT coding

Two middle and one high school science teachers’ 13 classroom observation videos were coded using CDAT to examine the research questions and to determine its reliability and validity as an instrument. The sample classroom videos are from a larger research project, Classroom Connectivity in Promoting Mathematics and Science Achievement (CCMS) funded by the Institute of Education Sciences, US Department of Education (grant R305K050045). The participants whose data were used in the present study constituted a subset of a larger pool (Science cohorts 3 and 4, *N* = 12) in the CCMS study. Only three physics teachers were selected out of 12 science teachers considering their completion of the project training, subject and grade level teaching, years of teaching experience, and the school locales (see Table 3). The samples were only selected for the pilot coding to see how the results are consistent with those by other observational instruments (i.e., TIR or EQUIP).

**Table 3:** Participant information

Teacher	State	Locale	Grade levels	Undergraduate major	Years teaching	Teaching subject	Observation videos
Ann	TX	Large city	7	Animal Science	4	Physics	4
Ben	PA	Urban fringe of a large city	7	Physics	2	Physics	5
Cory	OH	Mid-size city	10	Elementary Ed	16	Physics	4

All observation videos have the audio recorded and transcribed verbatim. Table 4 shows each teacher’s observed class time, discourse time on topic analyzed, numbers of dialogs coded, and average time per dialog.

**Table 4:** Comparison of each teacher’s classroom dialog time

Teacher	Total class time (h:m:s)	Discourse time on topic (h:m:s)	No. of dialogs	Average time per dialog
Ann	3:36:26	0:39:54	59	0:41
Cory	3:03:09	1:08:48	91	0:45
Ben	7:01:37	1:03:28	109	0:35

The CDAT coding results of the sample classroom discourses include each teacher’s (1) coded CDAT tables, (2) length of dialog (LOD), and (3) reasoning components used in a dialog. Since the purpose of this study (see RQ1 and RQ2 on page X) was to exam if CDAT coding identifies the characteristics of the dialogs between teachers and students in science classrooms, summative data of all discourse patterns are not presented in this present paper.

### (1) Coded CDAT tables: visualized descriptions of the classroom discourse

#### Teacher Ann

**Fig. 4:**
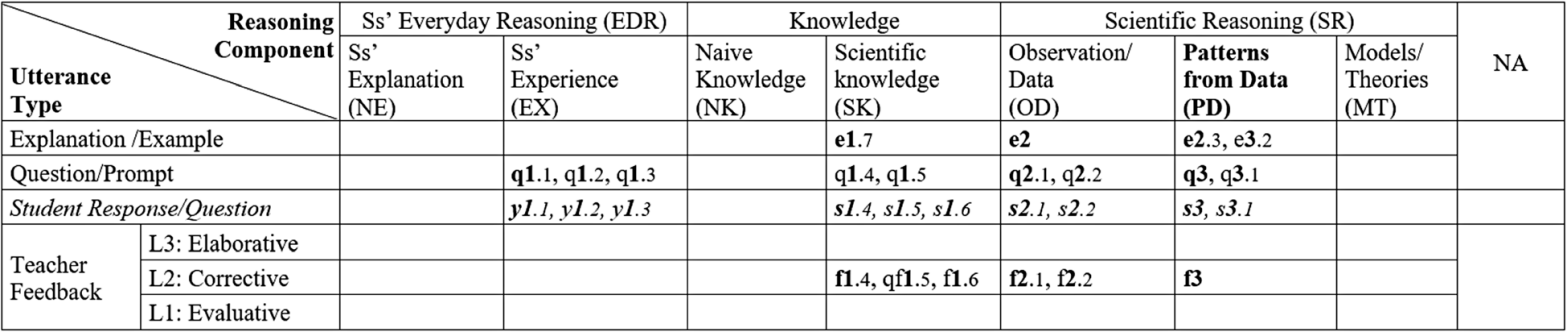
Ann’s CDAT table coded and the dialogs. At a glance, it shows what reasoning components the teacher used in the dialogs, i.e., EX, SK, OD, and PD. It also shows how the dialogs flow. It starts with a question about student experience (q1.1) and ends with an explanation (e3.2)

Figure 4 shows CDAT coding of three dialogs that are a part of Ann’s classroom discourse. The three dialogs are indicated by the numbers of *1*.*x*, *2*.*x*, and *3*.*x* in the table. At a glance, it shows the reasoning components of EX, SK, OD, and PD were used in the three dialogs. The distribution of codes in the table illustrates how the classroom discourse proceeded. Ann began the first dialog with questions about students’ experience (EX) related to the topic (q*1.1*, q*1.2*, and q*1.3*), and students responded with yes or no answers (*y1.1*, ***y****1.2*, and *y1.3*). Then, the teacher asks students about the concept of speed (q1.4 and q1.5), and students responded with short answers (s1.4, s1.5, and s1.6). It also shows the order of dialogs, starting with a question about student experience (EX) and ending an explanation about patterns from the data (PD). In addition, the levels of teacher feedback were assessed by the rubrics in Table 3. For instance, the feedback f1.4, “*it’s movement ok*,” is coded as level 2 because it is just a repeat of student’s response implying the answer is right. The feedback of qf*1.5*, “distance traveled over…,” is also coded as level 2 because it is a confirmation using a form of question asking students to finish the phrase. After this dialog, the discourse continued to the second dialog (numbered 2.*x*) started with an explanation about observation/data (OD) from their classroom activities and to the third dialog (numbered 3.*x*) about patterns from the data.

Dialogs coded in Fig. 4

T: Ok. So what is speed? You all should probably be able to tell me that without even having to look at your book because you speed to school every day, right? **q1.1**

SS: Nope. No. **y1.1**

T: You don’t? Don’t you all ride the school bus? **q1.2**

SS: Yes. No. **y1.2**

T: Is speed involved in getting to school? **q1.3**

S: Yeah. **y1.3**

T: Yeah, what is speed? **q1.4**

SS: Movement. **s1.4**

T: It’s movement, ok, **F1.4** and what two things do we use to calculate speed? **q1.5**

SS: Distance traveling over time. **s1.5**

T: Distance traveled over…**qf1.5**

SS: Time. **s1.6**

#### Teacher Ben

**Fig. 5:**
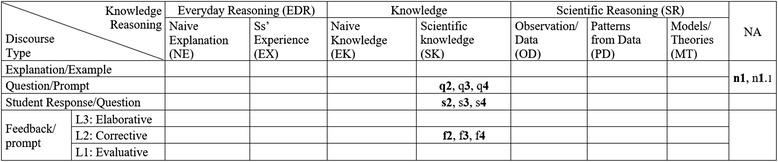
Ben’s CDAT table coded and the dialogs

The table in Fig. 5 describes Ben’s classroom discourse patterns. At a glance, it shows that the teacher only focused on scientific knowledge. The first two teacher’s utterances are coded in the cell NA with number “n1 and n1.1” because they are not relevant to the topic and can be considered as a classroom management talk. The dialogs q2, q3, and q4 do not have any follow-up questions or feedback after the first sequence of question, response, and feedback. The dialogs show typical QRF patterns, i.e., q2, s2, f2; q3, s3, f3; q4, s4, f4, coded in the cells of scientific knowledge column. Although some of his dialogs include additional student responses and teacher feedback, most of his dialogs are just simple QRF patterns with only level 1 and level 2 feedback.

Two coders pondered whether it should be considered as a continued dialog or a separate dialog when a teacher asks exactly the same question, but to another student. The coders eventually agreed that when a teacher provides a corrective or evaluative feedback such as “very good” or “correct,” then it can be considered that the dialog ends unless a follow-up question about the student’s response occurred. Thus, the dialogs of 2, 3, 4, and 5 in the discourse are considered as separate since they all end with a corrective feedback without any further questions to the students.

Dialogs coded in Fig. 5

T: Who wants to do catch? Question for Stephanie. **n1** Guys, as we go through the review, pay attention because everything you answer on

the sheet you can turn in at test time and get credit. **n1.1**

Alright? Stephanie your question is Number 8, what flows in the water? Number 8,

what flows in the water? **q2**

S: Electricity? I do not know. **s2**

T: Okay. Electricity would be correct so I cannot say it is wrong. **f2**

What flows in water, Curt? **q3**

S: Current. **s3**

T: Current very good! **f3**

Ed, what flows in the water? **q4**

S: Electrons. **s4**

T: Electrons, very good! **f4**

…

#### Teacher Cory

**Fig. 6:**
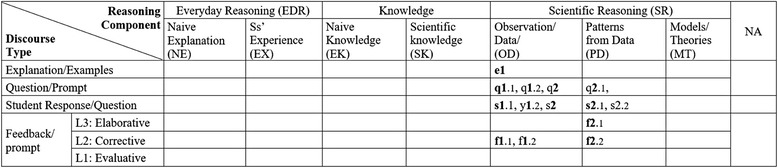
Cory’s CDAT table coded and the dialogs

The CDAT coding table in Fig. 6 shows Cory’s classroom discourse patterns. At a glance, it shows the two dialogs focused on OD and PD. She started the first dialog with an explanation about students’ observations and then asked follow-up questions followed by student responses with short answers (s1.1) and teacher feedback (f1.1). Then, she asked a follow-up question (q1.2) followed by students’ answers (y1.2). It shows a discourse pattern of QRFQRF. She started the second dialog with a question about another part on the graph (q2) followed by students’ short answer (s2.1). Then, her follow-up question is now about patterns from the data, q2.1 “Why does the line go straight?” instead of providing feedback. Her feedback of 2.1 was coded as level 3 because it prompted students to do a high-level thinking process, reasoning with evidence. The questions were answered by several different students (s2, s2.1, and s2.2). Cory’s length of dialogs (LOD) are typically larger than 3, which indicates they are beyond the simple QRF patterns.

Dialogs coded in Fig. 6

T: This is at the beginning when you were kind of going like that, so it messed up a little bit, but once you started walking in a straight line. **e1**

I didn’t want you to veer from that line – look what happened. What does that show? **q1.1**

SS: The slope. **s1.1**

T: That’s the slope. **f1.1**

Was she going very fast? **q1.2**

SS: No. **y1.2**

T: No; nice average speed. **f1.2**

What happened here? **q2**

SS: She stopped. **s2**

T: Why does the line go straight? **q2.1**

SS: Time keeps going. **s2.1**

T: Time keeps going but she’s not gaining any… **f2.1**

S: It’s going to go up because you don’t have to stop. **r2.2**

T: Alright, we’re going to see. **f2.2**

…

### (2) LOD and number of reasoning components used

Table 5 shows the numbers of each teacher’s dialogs coded, average dialog time, average numbers of reasoning components used in a dialog, and average LOD. The table shows specific information about the teachers’ classroom discourse patterns. For example, Ann’s average number of reasoning components used in a dialog is 1.46, which is higher than that of the other two teachers, so is her average length of dialogs (*M =* 4.69). While Ben’s average number of reasoning components used in his dialog is 1.05 and his average length of dialogs are less than 3 (*M =* 2.38). Cory’s average number of reasoning components used in a dialog is 1.40 that is relatively high, and her average LOD are longer than 4 (*M =* 4.15). Although we are not assuming any generalized conclusions, this results postulate that if teachers use more reasoning components, then their dialogs become longer with more students’ responses.

**Table 5:** Comparison of each teacher’s discourse patterns

	Total no. of dialogs	Average time per dialog	Average numbers of reasoning components	Average length of dialogs (LOD)
Ann	59	0:41	1.46	4.69
Cory	91	0:45	1.40	4.15
Ben	109	0:35	1.05	2.38

### 3) Reasoning components used in the classroom discourse

CDAT analysis also revealed teachers’ characteristics in the use of reasoning components. Although Ann used the reasoning component of scientific knowledge (SK) the most, she used a great amount of student experience (EX), observation and data (OD), and patterns from data (PD) throughout the classes analyzed. As shown in Fig. 4, she used more than three reasoning components (SK, OD, EX, or PD) in her dialogs coded. The patterns in movement on reasoning components in her dialogs were typically from scientific knowledge (SK) to student experience (EX) and from observation/data (OD) to patterns from data (PD). Likewise, Cory showed specific patterns of using the reasoning components. She typically started a dialog with an explanation and then gave a question about scientific knowledge followed by questions about observations/data and then about patterns from data. On the other hand, Ben mostly asked only about scientific knowledge and rarely used any other reasoning components. The patterns in the use of reasoning components in classroom discourse may not determine if the discourse is scientific or not, but it shows how teachers make connections among the reasoning components, i.e., deductively or inductively.

## Reliability and validity for the CDAT

### Reliability

Inter-rater reliability is typically defined as the measurement of the consistency between evaluators in the *ordering* or *relative standing* of performance ratings, regardless of the *absolute* value of each evaluator’s rating (Graham et al. 2012). Inter-rater agreement is the degree to which two or more evaluators using the same rating scale to give the same rating to an identical observable situation (e.g., a lesson, a video, or a set of documents). Thus, unlike inter-rater reliability, inter-rater agreement is a measurement of the consistency between the *absolute value* of evaluators’ ratings (Graham et al. 2012). For this study, inter-rater agreement is considered a better fit because the CDAT coding is more likely to reflect the teachers’ discourse characteristics rather than a rater’s opinion about the relative levels of performance. In this study, three indexes of inter-rater agreement were calculated, the percentage of absolute agreement, Cohen’s kappa, and the intra-class correlation coefficient using SPSS statistical package.

Prior to conducting observations of the classroom videos for coding, the author trained the other coder, a doctoral student in science education, to ensure that she understood the coding categories and interpreted the indicator utterances similarly. During this training, the two coders observed and coded one sample classroom video and compared their interpretations and coding using the CDAT categories of *utterance types* and *reasoning components*. Through following discussions, they arrived at a common understanding of the coding scheme and categories. Then, they coded the classroom discourse from three teachers’ classroom videos independently and calculated the three types of inter-rater agreement indexes.

All three inter-rater agreement indexes show acceptable to strong consistency among two coders (Table 6). The inter-rater agreement analyses confirmed that the CDAT coding is consistent irrespective of who is coding the classroom discourse. The agreement analyses were conducted at both the first step and the second steps of the CDAT coding; the first step is to determine types of a teacher/student utterance (i.e., question, feedback, explanation, response) and the second step is to determine which reasoning component the utterance belongs to (i.e., student experience, observation/data, patterns from data, theory/model).

**Table 6:** Inter-rater agreement indexes between two coders

Agreement statistic	First step coding	Second step coding	Recommended acceptable range
Intra-class correlation	.81	.78	.90–70
% Absolute agreement	94.6%	76.3%	90–75%
Cohen’s Kappa	.93	.70	.81–.61

Although, an intra-class correlation (ICC) is suggested since CDAT coding has more than 10 coding categories, Kappa and the percentage of absolute agreement were also calculated and compared to ICC scores. ICC represents the proportion of the variation in the coding that is due to the characteristics of the teacher being assessed rather than how the rater interprets the rubric. ICC scores generally range from 0 to 1, where 1 indicates perfect agreement and 0 indicates no agreement between one rater and another single rater (labeled “single measure” in the SPSS output). The single measure intra-class correlation shows the agreement among raters, and thus, how well an evaluation rating based on the ratings of one rater is likely to agree with ratings by another rater. Reliability studies also suggest that, when using percentage of absolute agreement, values from 75 to 90% demonstrate an acceptable level of agreement (Hartmann 1977; Stemler 2004). For Kappa, popular benchmarks for high agreement are .80, and minimum acceptable agreement are .61 (Altman 1990; Landis and Koch 1977). For ICC, typically, .70 would be sufficient for a measure used for research purposes (Hays and Revicki 2005; Shrout and Fleiss 1979).

### Validity

#### Construct validity: coding procedures and definitions of coding components

To build valid components of CDAT, the coding categories were developed based on the review of literature that relates to scientific/everyday reasoning and discourse. In addition, the definitions of each reasoning component were refined to help coders find the utterances that fit the categories. The category definitions and an actual transcript of a part of a teacher’s classroom discourse were sent to two science educators in two universities in the Midwest USA. They were asked to answer three questions. First, if the definitions are clearly defined and fit the current ideas in science education, second, if the coded utterance examples fit the categories as defined, and third, if the coded CDAT coding results represent the classroom discourse well without looking at the actual transcripts.

The two educators agreed that the definitions make sense and are clear enough to determine a teacher or student utterance as one of the coding components. One of them, however, pointed out that using the term “naïve” frames students’ ideas and resources as uninformed and perhaps in a deficit light. Further, he suggested using “everyday explanations” or “everyday knowledge” instead of “misconceptions” or “naïve” since this might imply a deficit perspective toward student resources as described in the definitions. This suggestion is also consistent with the literature reviewed in this paper in which “everyday reasoning” derives from valid and important everyday experiences and ways of knowing.

The expert panel members were also asked to provide their thoughts about (1) if the utterances coded as each category are well matched with its definition and (2) if the coded CDAT table describes the discourse well. They also agreed that the CDAT coded results well illustrate the teacher’s discourse patterns at some levels, but not entirely. One of them pointed out that some of the student responses are just confirmatory answers to the teacher’s questions or student agreement. The example episode shows several reasoning components on the teacher’s part, but not on the students’ part with very short answers mostly “yes.” This expert also expressed that the CDAT table might show something that comes across as richer than this episode really is. Thus, the expert suggested that the CDAT table could be strengthened by adding an element that measures the length and richness of student responses.

#### Concurrent validity: comparisons of CDAT with EQUIP and TIR

The results from CDAT coding were compared with those from two inquiry-based science classroom observation rubrics, Electronic Quality of Inquiry Protocol (EQUIP; Marshall et al. 2009) and Teacher Inquiry Rubric (TIR; Nugent et al. 2011). Since CDAT coding is not to evaluate a teacher’s discourse patterns but to describe them, possible interpretations of the results such as distribution of the reasoning components used, the patterns of teacher and student interaction, or the levels of teacher feedback were compared with the ratings of EQUIP and TIR by two former science instructional coaches. The instructional coaches were trained for the EQUIP and TIR assessments and have assessed almost 300 middle and high school science classroom observation videos in a 3-year IES funded project. The individual indicator and overall ratings of EQUIP and TIR by the two coaches were compared with the results of CDAT coding of the same teacher classroom discourse by the authors, to see if they supported the same interpretation of teachers’ discourse patterns. For a validity check, the two coaches’ ratings in each teacher’s video were compared with the results of CDAT coding.

The EQUIP ratings of Cory’s discourse were overall level 2.5 (between level 2 and 3) and level 3 by the two coaches. Particularly, the indicators of Communication Pattern and Classroom Interactions were all rated as level 3. The CDAT coding results showed the variety of reasoning components used by the teacher, which is consistent with the ratings by EQUIP (Table 7). While Ben and Ann’s discourse level were evaluated overall as level 1.3 and 1.8 by the two coaches, Ben’s CDAT discourse patterns (Fig. 5) showed limited number of reasoning components and simple IRE discourse patterns which might be consistent with this evaluation. However, although Ann’s CDAT coding results could be interpreted as a higher level of discourse, the EQUIP ratings by the coaches are lower than expected by CDAT. The coaches pointed out that Ann’s questions were mostly to check for understanding and correct responses. Her patterns in the CDAT table show some varieties among the reasoning components and a good number of student responses, but it did not reveal the quality of teacher questions and student responses, i.e., yes or no or open-ended questions.

**Table 7:** Comparisons between the three methods (EQUIP, TIR, and CDAT)

Teacher	CDAT		EQUIP	TIR
	Focused reasoning components	Discourse pattern (LOD)	Discourse level	Level of communication
Ann	SK, EX, OD, PD (4)	Extended QRF (4.7)	1.8	1
Cory	SK, OD, PD (3)	Extended QRF (4.2)	2.5	3
Ben	SK (1)	Simple QRF (2.4)	1.3	1.5

The *Teacher Inquiry Rubric* (TIR) is a four-level rubric assessing teacher proficiency in guiding students to develop necessary skills in science questioning, investigating, explaining, communicating, and applying science knowledge to a new situation (Nugent et al. 2011). Within each of the six constructs, there are four levels: “pre-inquiry,” “developing inquiry,” “proficient inquiry,” and “exemplary inquiry.” For a validity check, only the evaluation of communicating construct was compared with the CDAT coding results. Cory’s level of communication by TIR rubrics was rated as level 3 by the two coaches. The coaches also pointed out the teacher used guiding questions and helped students understand the data. Ben’s communication level rated by TIR is level 1.5 (between pre-inquiry and developing inquiry) that is consistent with CDAT coding results (Fig. 5) with extremely limited numbers of reasoning components and simple IRE discourse patterns. The coaches also made a note that the teacher provided most of the questions, and students’ responses were very limited. However, Ann’s communication level by TIR was assessed as level 1 that is not consistent with the CDAT coding results (Fig. 4). Again, this result shows that CDAT coding does not provide information about the quality of teacher questions and students’ answers, but rather CDAT describes the patterns.

## Discussion

**Fig. 7:**
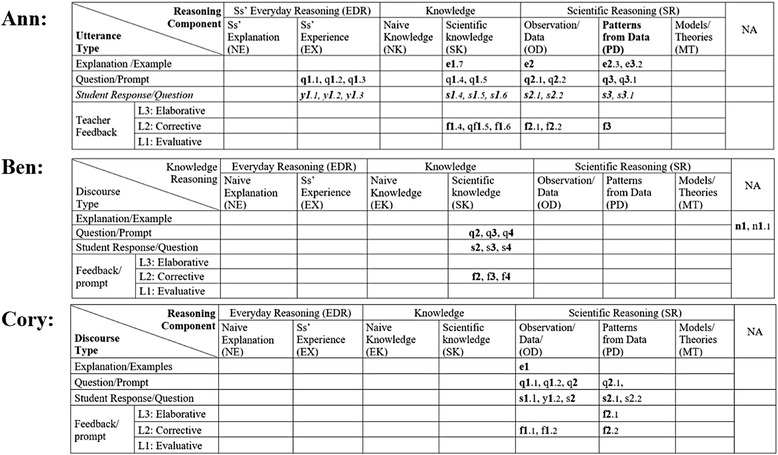
Comparison of three different teachers’ CDAT coding tables

The Classroom Discourse Analysis Tool (CDAT) is not meant to evaluate teachers’ classroom discourse, but to reveal (a) teachers’ discourse patterns with the lens of scientific reasoning and inquiry and (b) levels of students’ engagement in the classroom discourse. For this study, the research questions asked how well CDAT coding results disclose the patterns and characteristics of a teacher’s classroom discourse. The CDAT suggests a new discourse pattern finding with the two-dimensional graphic organizer and the data produced by the coding. A coded CDAT table shows at a glance what reasoning components used in the classroom dialogs between the teacher and students. It is more distinct when they are compared side by side (Fig. 7). It also shows how students engaged in the dialogs with the variations of their answers by the teacher’s question and feedback. As it shown in the tables, the patterns of students’ responses strongly depend on teacher’s question or feedback with almost no exceptions. In addition, this analysis also generated various quantitative data that represent certain characteristics of the classroom discourse, i.e., length of dialog and the number of reasoning components used.

Unlike the Likert-based observation protocols that require coders to make holistic judgments to rate broad categories of a teacher’s instruction, CDAT coding is rather to determine teacher’s or students’ utterance types throughout the class. A coder is not needed to judge the teacher’s level of each aspect of the classroom instruction. Although it was designed to assess classroom discourse in relation to scientific reasoning and formative feedback, the components in CDAT are not directly associated with a specific type of instruction (i.e., inquiry-based or constructivist’s). What the coded CDAT tables offer are not evaluations or opinions but a collected data set that describes objectively the classroom dialogs. Using the data from CDAT tables, teachers will be able to see (1) which reasoning components were used or not, (2) how they are connected through the dialogs, (3) how well students were engaged in the dialogs, (4) if the dialogs are extended IREs, and (5) what levels of feedback were used. The data from the tables can be used for a data-driven instructional coaching if a teacher needs an interpretation that will assist in improving the teachers’ instructional practice (Lee et al., 2014b).

The presented study provides acceptable levels of evidence of reliability and validity for CDAT coding components and process. Although the reliability check was conducted between only two coders, three different types of reliability indexes showed all moderate to substantial agreement. However, further reliability checks of the CDAT with more coders conducted with more science classroom discourse are needed to further verify the reliability. Overall, the reliability and validity analyses conducted for the CDAT coding demonstrate acceptable and consistent evidence of the instrument’s usefulness in documenting the characteristics of a teacher’s classroom discourse. Although the presented study did not involve the instrument’s use by teachers, with appropriate training, the instrument could be a valuable resource in assessing the presence or absence of essential indicators for scientific discourse.

Various science classroom observational protocols have been developed and used, such as Electronic Quality of Inquiry Protocol (EQUIP) or Reformed Teaching Observation Protocol (RTOP) (Marshall et al. 2009; Piburn et al. 2000). These tools, however, use Likert-type assessments for one whole-class period. Compared to other instruments, with the finer grained CDAT coding, teachers are offered information that is specific to their practice of classroom discourse with regard to both the level of scientific reasoning and student engagement. Although some decisions are also demanded of coders, they are not overall evaluations, but rather data-collecting procedures. The accumulated data from the CDAT coding generates overall descriptive information such as average length of dialog, what and how reasoning components used, and ratio of teacher utterances and students’.

## Conclusion

The results from the assessment of three sample classroom discourses using EQUIP and TIR by the coders mostly agreed with the CDAT coding results. However, there is some disagreement on describing the quality of the classroom discourse. For example, although one teacher’s discourse showed various distributions of the reasoning components in the coded CDAT table, the EQUIP and TIR assessment results pointed out that her questions were mostly to check for student understanding and correct responses. Thus, a possible modification of the CDAT for future use might include categories to assess the level of teachers’ questions and student responses. Future work is also needed to further support the validity and reliability of the CDAT instrument. First, to build predictive validity, the relationship between the disclosed teachers’ discourse patterns by CDAT coding and students’ learning outcomes needs to be studied. Second, to disclose more types of classroom discourse, the teacher’s dialogs with individual students or a small group need to be included in CDAT coding to fully represent the classroom discourse.

The most possible implications of CDAT analysis is, first, to explore the relationships between teachers’ discourse patterns and students’ achievement along with changes in their reasoning skills. Student attitudinal outcomes such as motivations, interests, or self-efficacy could also be compared by the classroom discourse patterns revealed by CDAT. CDAT coding can also be used in a professional development as an intervention to help teachers see their classroom discourse patterns. Second, with the quantitative data produced by CDAT analysis (i.e., LOD, the numbers of reasoning components used, levels of teacher feedback), various relationships between teachers’ discourse patterns and other intended outcomes in a PD project. Third, it can also be used to characterize discourse among students. Diverse types of student-student talk exist in science classrooms, such as small group talk, presentations, and argumentation practice. Lastly, CDAT can also be used for students’ written explanations to examine which reasoning components they choose and how they use them through the phrases or sentences they include in their writings.

## Additional files

Additional file 1: Quantitative data of the classroom dialogues analyzed in the study. (XLSX 279 kb)

Additional file 2: The entire CDAT coding tables used in the study. (PDF 269 kb)
