# Supplementary material for: Development of Two-Dimensional Classroom Discourse Analysis Tool (CDAT): scientific reasoning and dialog patterns in the secondary science classes
Source: Int J STEM Educ. 2018 Feb 19;5(1):5. doi: 10.1186/s40594-018-0100-0 (PMC6310396; doi:10.1186/s40594-018-0100-0)
Supplement: Supplementary file 2 — The entire CDAT coding tables used in the study. (PDF 269 kb) [file 40594_2018_100_MOESM2_ESM.pdf]

03026NM-2007-04-26 p01 Ep1.1 w/o CCT [18:20 ~ 20:25]

Knowledge Reasoning / **Discourse Type**		Ss' Everyday Reasoning (EDR)		Knowledge		Scientific Reasoning (SR)			N/A
		Ss' Explanation (NE)	Ss' Experience (EX)	Naive Knowledge (NK)	Scientific knowledge (SK)	Observation/ Data (OD)	**Patterns from Data (PD)**	Models/ Theories (MT)	
Explanation /Example					1.7	2,2.1,2.2	2.4,3.2		
Question/Prompt			1.1,1.2,1.3		1,1.4,1.5	2.3,2.4	3,3.1		
Student Response/Question			1.2,1.3		1.4,1.5,1.6	2.3,2.4	3,3.1		1.1
Feedback/ prompt	L3:Elaborative								
	L2:Corrective				1.4,q1.6,1.6	2.3,2.4	3		
	L1:Evaluative								

* Topic: Speed, Type: Lecture, CCT: No
* # of Dialogs: 3, **length of D**: 11, 4, 4, # of Reasoning Areas: (SK,EX) (OD,PD) (PD)
* # of Utterances: 33, teacher's: 22, students': 10, # of N/A:1
* # of T's Utterances in: EX: 3, SK:7, OD:7, PD:7, E: 6, Q:10, Feedback: 5, L1:0, L2:5, L3:0,
* # of Ss' Questions: 0

3026NM-2007-04-26 p01 Ep1.2 w/o CCT [20:26 ~ 22:59]

Knowledge Reasoning / Discourse Type		Ss' Everyday Reasoning (EDR)		Knowledge		Scientific Reasoning (SR)			N/A
		Ss' Explanation (NE)	Ss' Experience (EX)	Naïve Knowledge (EK)	Scientific knowledge (SK)	Observation/ Data (OD)	Patterns from Data (PD)	Models/ Theories (MT)	
Explanation /Example			3.7,3.8,3.8		3,3.1,3.5	1.6,1.7,1.8,1.9 ,1.10,1.11,1.1 2,1.13	2.5		1.2,1.5
Question/Prompt			2,2.1,2.4		1,1.4,3.2,3.4,3.6	1.1,1.3			
Student Response/Question			2.1,2.2,2.4		1.4,1.5,3.2,3.3,3.4,3.6				
Feedback/ prompt	L3:Elaborative		q2.2,q2.3						
	L2:Corrective		2.4		q1.5,q3.3,3.4,3.6				
	L1:Evaluative								

* Topic: Average speed vs. Instantaneous speed, Type: Lecture, CCT: No
* # of Dialogs: 3, **length of D**: 3, 6, 6, # of Reasoning Areas: (SK,OD) (EX,PD), (SK,EX)
* # of Utterances: 46, teacher's: 35, students': 9, # of N/A:2
* # of T's Utterances in: EX: 12, SK:18, OD:11, PD:1 / E:15, Q: 10, Feedback: 7, L1:0, L2:5, L3:2,
* # of Ss' Questions: 0

3026NM-2007-04-26 p01 Ep02 with CCT [52:04 ~ 54:25] After giving about 30 minutes for LC

Knowledge Reasoning / Discourse Type		Ss' Everyday Reasoning (EDR)		Knowledge		Scientific Reasoning (SR)			N/A
		Ss' Explanation (NE)	Ss' Experience (EX)	Naïve Knowledge (EK)	Scientific knowledge (SK)	Observation/ Data (OD)	Patterns from Data (PD)	Models/ Theories (MT)	
Explanation /Example					1.9	1.14	2.2,2.3,2.4		1,1.1,1.2,1.6,1.7,1.15
Question/Prompt					1,1.8	1.10,1.11,1.12,1.13,1.16,1.17,1.18,2,2.1			
Student Response/Question					cR1,	1.11,1.12,1.17,1.18,1.19,2.1			1.7
Feedback/ prompt	L3:Elaborative					q1.19			1.3,1.5
	L2:Corrective				1.4	1.11,1.12,2.1			
	L1:Evaluative					1.17			

* Topic: What equilibrium meant, Type: Lecture (After 30m PS), CCT: LC, showing students' responses on the screen.
* # of Dialogs: 2, **length of D**: 11, 2, # of Reasoning Areas: (SK,OD) (OD,PD)
* # of Utterances: 47, teacher's: 30, students': 8, # of N/A:9
* # of T's Utterances in: EX: 0, SK:5, OD:20, PD:3 / E:5, Q: 11, Feedback: 6, L1:1, L2:4, L3:1
* # of Ss' Questions: 0

3026NM-2007-04-26 p01 Ep03 with CCT [54:30 ~ 56:46]

Knowledge / Reasoning — Discourse Type		Ss' Everyday Reasoning (EDR)		Knowledge		Scientific Reasoning (SR)			N/A
		Ss' Explanation (NE)	Ss' Experience (EX)	Naïve Knowledge (EK)	Scientific knowledge (SK)	Observation/ Data (OD)	Patterns from Data (PD)	Models/ Theories (MT)	
Explanation /Example					4.2,6.1				
Question/Prompt					1,1.3,1.4,1.5,2,2.1,2.2,2.3,3,5.2,6,7,8				1.1,2.5,5.3
Student Response/Question					cR,1.3,1.4,1.5,2,2.1,2.2,2.3,3,5.1,5.2,6,7,8				
Feedback/ prompt	L3:Elaborative				q5.1				
	L2:Corrective				1.4,1.5,2,2.1,2.2,2.3,3,4,4.1,5,5.2,6,7,8				2.4,4
	L1:Evaluative								

* Topic: climax community, Type: Lecture, CCT: LC, showing students' responses on the screen. 1 Class response
* # of Dialogs: 8, **length of D**: 8,8,2,1,4,2,2,2 # of Reasoning Areas: all (SK)
* # of Utterances: 49, teacher's: 30, students': 14, # of N/A:5
* # of T's Utterances in: EX: 0, SK:44, OD:0, PD:0 / E:2, Q: 13, Feedback: 15, L1:0, L2:14, L3:1
* # of Ss' Questions: 0

3026NM-2007-04-26 P3 Ep01 CCT [13:20 ~ 19:25] the same content with previous one but different class

Knowledge Reasoning / **Discourse Type**		Everyday Reasoning (EDR)		Knowledge		Scientific Reasoning (SR)			N/A
		Ss' Explanation (NE)	Ss' Experience (EX)	Naive Knowledge (EK)	Scientific knowledge (SK)	Observation/ Data (OD)	Patterns from Data (PD)	Models/ Theories (MT)	
Explanation/Examples			3.3,3.4,3.5		3.2,5.9,6.1, 6.2,9.6	2.3,5,5.1,7.6,7 .7,7.8,7.12,7.1 3,7.14,	5.5,5.6,5.7,5. 8,7.17		7.3,9
Question/Prompt			2,2.1,2.2,9.1,9 .3,9.5		1,3,3.1,4,6, 7,7.1,7.2,7. 3,9,10	5.2,5.3,7.9,7.1 5,9.4	7.16,8		
Student Response/Question			2,2.2,9.2,9.3,9 .5		1,3.1,4,6,7. 3,7.4,7.5,9, 10,10.1	5.2,5.3,5.4,7.8 ,7.9,7.10,7.11, 9.4	7.16,8		2.1
Feedback/ prompt	L3:Elaborative				q7.5	q5.4,q7.10,	8		
	L2:Corrective		q2.1,9.2,9.3,9. 5		1,3.1,6,9,10 ,10.1	7.10,q7.11,7.1 1,9.4	7.16,		
	L1:Evaluative								

* Topic: Speed, Average vs. Instantaneous Speed, Type: Lecture, CCT: No
* # of Dialogs: 10, **length of D**: 2,3,2,1,6,3,12,2,10,4 # of Reasoning Areas: (SK) (EX,OD) (EX,SK) (SK) (OD,PD) (SK) (OD,SK,PD) (PD) (SK,EX) (SK)
* # of Utterances: 93, teacher's: 64, students':26, N/A:3
* # of T's Utterances in: EX: 18, SK:32, OD:28, PD:11, E:22, Q:24, Feedback: 19, L1:0, L2:15, L3:4,
* # of Ss' Questions: 0

3026NM-2007-04-26 P3 Ep02 CCT [45:37 ~ 51:52] the same content with previous one but different class

**Discourse Type** \ Knowledge Reasoning		Everyday Reasoning (EDR)		Knowledge		Scientific Reasoning (SR)			N/A
		Ss' Explanation (NE)	Ss' Experience (EX)	Naive Knowledge (EK)	Scientific knowledge (SK)	Observation/ Data (OD)	Patterns from Data (PD)	Models/ Theories (MT)	
Explanation/Examples			1.8		1.5,1.9,2.4, 4.5,6.2,6.3, 6.4,6.5,8.3, 8.6,9.2,9.3, 9.4,13	1.6,1.7,7.1,7.2			1.2,2.1,2.2, 7.3
Question/Prompt					1,1.1,2,2.3, 3,3.1,4,4.1, 4.2,4.3,4.4, 5,8,8.4,9,9. 1,10,11,11. 1,11.2,12,1 2.1,13,14	7			
Student Response/Question					cR,1.4,2.3, 3.1,4,4.1,4. 2,4.3,4.4,5, 6.1,6.3,8,8. 3,8.4,9,9.1, 9.4,10,11,1 1.1,11.2,12, 12.1,13,14	7			2.1
Feedback/ prompt	L3:Elaborative				q1.4,1.4,q6. 1				
	L2:Corrective				2.3,3.1,4,4. 1,4.3,4.4,5, 6,6.1,8,8.4, 10,10.1,11. 2,12,12.1,1 3				1.3,2.1
	L1:Evaluative				8.1,8.2,8.5				

* Topic: Equilibrium, Climax Community, Type: Lecture, CCT: LC showing students' responses
* # of Dialogs: 14, **length of D**: 3,2,2,8,2,3,2,6,5,2,5,4,2,1 # of Reasoning Areas: (SK,OD,EX) All (SK) except 7 (OD)
* # of Utterances: 107, teacher's: 73, students':27, N/A:7
* # of T's Utterances in: EX: 1, SK:93, OD:6, PD:0, Q:24, E: 14, Feedback: 23, L1:3, L2:17, L3:3
* # of Ss' Questions: 0

3026SW-2007-04-27p1 Ep01 w/o CCT [12:16 ~ 15:18]

Knowledge Reasoning / **Discourse Type**		Everyday Reasoning (EDR)		Knowledge		Scientific Reasoning (SR)			N/A
		Ss' Explanation (NE)	Ss' Experience (EX)	Naive Knowledge (EK)	Scientific knowledge (SK)	Observation/ Data/ (OD)	Patterns from Data (PD)	Models/ Theories (MT)	
Explanation/Examples					1,2.1,2.2,5. 1,5.6,5.7	1.1,3.4,3.5,5	3.6		2.3,3.1,3.7, 3.8,3.9,4.1, 4.2
Question/Prompt					1.2,2,3,3.2, 4,5,5.2				
Student Response/Question					1.2,2,3.2,3. 3,4,5.2,5.3, 5.4,5.5				4.2
Feedback/ prompt	L3:Elaborative				q3.3,q5.3,5. 4,q5.5				
	L2:Corrective				1.2,2,3.3,4, 5.3,5.5				
	L1:Evaluative								

* Topic: Speed, Constant speed, Acceleration, Type: Lecture/Review, CCT: NO
* # of Dialogs: 5, **length of D**: 2,2,4,2,6 # of Reasoning Areas: (SK,OD) (SK) (SK,OD,PD), (SK), (SK,OD)
* # of Utterances: 35, teacher's: 25, students': 10, # of N/A:8
* # of T's Utterances in: EX:0, SK:32, OD:4, PD:1, E:11, Q:7, Feedback: 10, L1:0, L2:6, L3:4
* # of Ss' Questions: 0

3026SW-2007-04-27p1 Ep02 w CCT [23:28 ~ 24:45] after lab watching the students' works on the screen

Knowledge Reasoning / **Discourse Type**		Everyday Reasoning (EDR)		Knowledge		Scientific Reasoning (SR)			N/A
		Ss' Explanation (NE)	Ss' Experience (EX)	Naive Knowledge (EK)	Scientific knowledge (SK)	Observation/ Data/ (OD)	Patterns from Data (PD)	Models/ Theories (MT)	
Explanation/Examples						1.3,1.13,1.18			1,1.1,1.2,1.12
Question/Prompt					1.8,1.9,1.10	1.4,1.5,1.6,1.14,1.17,1.19			
Student Response/Question					1.10	1.4,1.5,1.6,1.7,1.14,1.15,1.16,1.17,1.19			
Feedback/ prompt	L3:Elaborative					q1.11			
	L2:Corrective				1.10	1.6,1.14,1.15,1.17,1.19			
	L1:Evaluative								

* Topic: Speed/ Graph, Type: Demonstration, CCT: Activity Center/ Graph/ Motion Detector
* # of Dialogs: 1, **length of D**: 16, # of Reasoning Areas: (OD,SK)
* # of Utterances:33, teacher's:19, students':10, N/A:4
* # of T's Utterances in: EX:0, SK:5, OD:24, PD:0, E:3, Q:9, Feedback: 7, L1:0, L2:6, L3:1
* # of Ss' Questions: 0

3026SW-2007-04-27p1 Ep03 w CCT [37:23 ~ 41:21] after lab watching the students' works on the screen

Discourse Type \ Knowledge Reasoning		Everyday Reasoning (EDR)		Knowledge		Scientific Reasoning (SR)			N/A
		Ss' Explanation (NE)	Ss' Experience (EX)	Naive Knowledge (EK)	Scientific knowledge (SK)	Observation/ Data/ (OD)	Patterns from Data (PD)	Models/ Theories (MT)	
Explanation/Examples					8.2,8.3,8.5	5.1,	8.6,8.7,8.8		1.1,1.2
Question/Prompt					8.4	1.3,1.4,1.5,1.6,2,2.1,2.2,2.3,3,4,5,7,7.1,7.2	6,6.1,6.5,8,		
Student Response/Question					cR,8.4	2.2,3,4,5,7.2	6.1,6.2,6.3,6.4,6.5,8,8.1		1.7,1.8,1.9,1.10,1.11,1.12,1.13,1.14
Feedback/ prompt	L3:Elaborative					q4.1	q6.2,q8.1		1.7,1.8,1.9,1.10,1.11
	L2:Corrective				8.4	1,2.2,3,4,4.1,5,7.2	q6.3,8.1		
	L1:Evaluative								

* Topic: Speed/Graph, Type: Review after Lab, CCT: Screen Capture/ Graph
* # of Dialogs:8, **length of D**: 2,3,2,2,2,6,2,6, # of Reasoning Areas: all OD but 6 (OD,PD) 8 (SK,PD)
* # of Utterances:66, teacher's:38, students':13, N/A:15
* # of T's Utterances in: EX:0, SK:6, OD:27, PD:18, E:7, Q:18, Feedback: 13, L1:0, L2:10, L3:3
* # of Ss' Questions: 0

3026NM-2007-04-27p3 Ep01 w CCT [12:35 ~ 15:55] watching the students' responses on the screen.

Knowledge / Reasoning — **Discourse Type**		Everyday Reasoning (EDR)		Knowledge		Scientific Reasoning (SR)			N/A
		Ss' Explanation (NE)	Ss' Experience (EX)	Naive Knowledge (EK)	Scientific knowledge (SK)	Observation/ Data/ (OD)	Patterns from Data (PD)	Models/ Theories (MT)	
Explanation/Examples					1.2,1.3,1.4, 2.2,2.5,3.2	2.3,2.7,2.10			1,1.1,2.4
Question/Prompt					1,2,3	2.6,2.8,2.9,3.3			
Student Response/Question					1.1,2,2.1,3, 3.1	2.6,2.8,2.9,3.3			1
Feedback/ prompt	L3:Elaborative				q2.1				
	L2:Corrective				1.1,2.1,q3.1 ,3.1	2.6,2.9,3.3			
	L1:Evaluative								

* Topic: Speed/Graph, Type: Review before Lab, CCT: No
* # of Dialogs:3, **length of D**: 2,10,6, # of Reasoning Areas: (SK) (SK,OD) (SK,OD)
* # of Utterances:37, teacher's:25, students':8, N/A:4
* # of T's Utterances in: EX:0, SK:19, OD:14, PD:0, E:9, Q:7, Feedback: 8, L1:0, L2:7, L3:1
* # of Ss' Questions: 0

3026NM-2007-04-27p3 Ep02 w CCT [50:30 ~ 54:10] watching the students' responses on the screen.

Knowledge Reasoning / **Discourse Type**		Everyday Reasoning (EDR)		Knowledge		Scientific Reasoning (SR)			N/A
		Ss' Explanation (NE)	Ss' Experience (EX)	Naive Knowledge (EK)	Scientific knowledge (SK)	Observation/ Data/ (OD)	Patterns from Data (PD)	Models/ Theories (MT)	
Explanation/Examples									
Question/Prompt					2.1	1.4,1.5,1.6,1.7,1.8,1.9,1.10,1.11,1.12,1.13,1.16,1.17,2.11,2.13	1.14,1.15,2,2.3,2.5,2.6,2.9,2.12,2.15,2.16		1,1.1,1.2,2.7,2.8,2.10,2.14
Student Response/Question					2.1,2.2	1.4,1.7,1.8,1.9,1.10,1.11,1.12,1.13,1.16,1.17,2.11,2.15	1.14,1.15,2,2.3,q2.4,2.12,2.16,2.17		
Feedback/ prompt	L3:Elaborative				q2.2		q2.17		
	L2:Corrective				2.2	1.7,1.8,1.12,1.13,1.16,2.11,2.15	1.14,1.15,2.3,2.17		
	L1:Evaluative					1.3			

* Topic: Speed/Graph, Type: Review after lab, CCT: Screen capture/Graph/Motion Detector
* # of Dialogs:2, **length of D**: 22,14 # of Reasoning Areas: (OD,PD) (PD,SK)
* # of Utterances:68, teacher's:40, students':21, N/A:7
* # of T's Utterances in: EX:0, SK:5, OD:34, PD:23, E:0, Q:26, Feedback: 15, L1:1, L2:12, L3:2
* # of Ss' Questions: 1

3033DB-2007-04-12 p01 Ep01 w/o CCT [10:54 ~ 13:36]

<table>
<tr><th colspan="2" rowspan="2">Knowledge Reasoning / Discourse Type</th><th colspan="2">Everyday Reasoning (EDR)</th><th colspan="2">Knowledge</th><th colspan="3">Scientific Reasoning (SR)</th><th rowspan="2">N/A</th></tr>
<tr><th>Ss' Explanation (NE)</th><th>Ss' Experience (EX)</th><th>Ss' ED Knowledge (EK)</th><th>Scientific knowledge (SK)</th><th>Observation/ Data (OD)</th><th>Patterns from Data (PD)</th><th>Models/ Theories (MT)</th></tr>
<tr><td colspan="2">Explanation/Examples</td><td></td><td></td><td></td><td></td><td></td><td></td><td></td><td rowspan="2">1,1.1</td></tr>
<tr><td colspan="2">Question/Prompt</td><td></td><td></td><td></td><td>2,3,4,5,6,7,8</td><td></td><td></td><td></td></tr>
<tr><td colspan="2">Student Response/Question</td><td></td><td></td><td></td><td>2,3,4,5,6.1,6.2,7,7.1,7.2,8</td><td></td><td></td><td></td><td>6</td></tr>
<tr><td rowspan="3">Feedback/ prompt</td><td>L3:Elaborative</td><td></td><td></td><td></td><td></td><td></td><td></td><td></td><td rowspan="3"></td></tr>
<tr><td>L2:Corrective</td><td></td><td></td><td></td><td>2,3,4,5,q6.2,6.2,q7.1,7.2,8</td><td></td><td></td><td></td></tr>
<tr><td>L1:Evaluative</td><td></td><td></td><td></td><td>6.1,7.3</td><td></td><td></td><td></td></tr>
</table>

* 5/6 What is the same question to other students? Not a feedback, not a sub question, ... a same question before getting a student's response could be n/a that is to get students' attention but a same question after getting students' responses and asking to other students is a new question with new numbers since it's not a sub, feedback, example, rephrase question but it related to the previous question.
* 5/6 Vague, just repeating students' responses are L1 feedback.

3033DB-2007-04-12 p01 Ep02 w/o CCT [14:30 ~ 14:47]

<table>
<tr><th colspan="2" rowspan="2">Knowledge Reasoning / Discourse Type</th><th colspan="2">Everyday Reasoning (EDR)</th><th colspan="2">Knowledge</th><th colspan="3">Scientific Reasoning (SR)</th><th rowspan="2">N/A</th></tr>
<tr><th>Ss' Explanation (NE)</th><th>Ss' Experience (EX)</th><th>Ss' ED Knowledge (EK)</th><th>Scientific knowledge (SK)</th><th>Observation/ Data (OD)</th><th>Patterns from Data (PD)</th><th>Models/ Theories (MT)</th></tr>
<tr><td colspan="2">Explanation/Examples</td><td></td><td></td><td></td><td></td><td></td><td></td><td></td><td rowspan="2">2</td></tr>
<tr><td colspan="2">Question/Prompt</td><td></td><td></td><td></td><td>1</td><td></td><td></td><td></td></tr>
<tr><td colspan="2">Student Response/Question</td><td></td><td></td><td></td><td>1,1.1,1.2</td><td></td><td></td><td></td><td>2,2.1</td></tr>
<tr><td rowspan="3">Feedback/ prompt</td><td>L3:Elaborative</td><td></td><td></td><td></td><td></td><td></td><td></td><td></td><td rowspan="3">2</td></tr>
<tr><td>L2:Corrective</td><td></td><td></td><td></td><td>1,q1.1,1.2,2.1</td><td></td><td></td><td></td></tr>
<tr><td>L1:Evaluative</td><td></td><td></td><td></td><td>q1.2</td><td></td><td></td><td></td></tr>
</table>

3033DB-2007-04-12 p01 Ep03 w/o CCT [15:16 ~ 17:26]

Knowledge / Reasoning — Discourse Type		Everyday Reasoning (EDR)		Knowledge		Scientific Reasoning (SR)			N/A
		Ss' Explanation (NE)	Ss' Experience (EX)	Ss' ED Knowledge (EK)	Scientific knowledge (SK)	Observation/ Data (OD)	Patterns from Data (PD)	Models/ Theories (MT)	
Explanation/Examples									
Question/Prompt					1,2				
Student Response/Question					1,2,2.1				1.1
Feedback/ prompt	L3:Elaborative								
	L2:Corrective								1.1
	L1:Evaluative				1				

* 5/6 A new question is not a feedback, a responding, an uptake, a sub, an example, that is, all responding students' previous answers is not a new question and
***** **5/6 All the questions that are not responding students' answers is a new question including the same question, rephrase, sub, and asking an example with new numbers not adding numbers after point.**

3033DB-2007-04-12 p01 Ep04 w/o CCT [17:35 ~ 18:52]

Knowledge / Reasoning — **Discourse Type**		Everyday Reasoning (EDR)		Knowledge		Scientific Reasoning (SR)			N/A
		Ss' Explanation (NE)	Ss' Experience (EX)	Ss' ED Knowledge (EK)	Scientific knowledge (SK)	Observation/ Data (OD)	**Patterns from Data (PD)**	Models/ Theories (MT)	
Explanation/Prompt/Example									
Question					**1,1-1,3,4**				
Student Response/Question					*1(2),1-1,q2,3,4,f4*				
Feedback/ prompt	L3:Elaborative								
	L2:Corrective				1-1,3,4				
	L1:Evaluative								

* Time of the Episode: sec, Average Teachers' Response Time < 1 sec, without CCT
* Teacher's Explanation with EX&EK (#4,#5) is not right
* All the student responses are brief with one word. Not a sentence

3033DB-2007-04-12 p01 Ep05 w/o CCT [19:05 ~ 21:10]

Discourse Type \ Knowledge Reasoning		Everyday Reasoning (EDR)		Knowledge		Scientific Reasoning (SR)			N/A
		Ss' Explanation (NE)	Ss' Experience (EX)	Ss' ED Knowledge (EK)	Scientific knowledge (SK)	Observation/ Data (OD)	**Patterns from Data (PD)**	Models/ Theories (MT)	
Explanation/Prompt/Example									
Question					**1,2,3,4,5,5-1,6,7,8**				
Student Response/Question					*1,2,3,4,5,5-1,5-2,5-3,5-4,6,7,8(2)*				
Feedback/ prompt	L3:Elaborative								
	L2:Corrective				1,2,3,4,5-1,5-2,5-3,8				
	L1:Evaluative								

* Time of the Episode: sec, Average Teachers' Response Time < 1 sec, without CCT
* Teacher's Explanation with EX&EK (#4,#5) is not right
* All the student responses are brief with one word. Not a sentence

3033DB-2007-04-12 p01 Ep06 w/o CCT [21:15 ~ 27:47] (many students' off topic talk)

Discourse Type \ Knowledge Reasoning		Everyday Reasoning (EDR)		Knowledge		Scientific Reasoning (SR)			N/A
		Ss' Explanation (NE)	Ss' Experience (EX)	Ss' Knowledge (EK)	Scientific knowledge (SK)	Observation/ Data (OD)	**Patterns from Data (PD)**	Models/ Theories (MT)	
Explanation/Examples			5.15		7.1,8.3,8.4,9.3,9.4,9.6				1,2,3.2,5,5.1,5.4,5.10,6.1,6.2,6.3,6.4,8.2,9.5
Question/Prompt			5.12,5.13,5.16		2.1,2.2,3,3.1,3,3.4,4,6,7,7.2,7.5,8,8.1,8.5,9	5.2,5.3,5.5,5.8,5.9,			
Student Response/Question			5.13,5.16,5.17,5.18		2.2,3.3,4.1,6,7,7.2,7.3,7.5,8,8.5,9,9.1,9.2	5.4,5.5,5.12			1,3.4,4,4.2,.6,5.10,5.11,5.14,6.2,7.4,8.1,8.2
Feedback/ prompt	L3:Elaborative							5.7	
	L2:Corrective		5.18,5.19		2.2,3.3,4.1,7.2,q9.1,9.1,9.2				1
	L1:Evaluative				7.3	.5			

* Time of the Episode: sec, Average Teachers' Response Time < 1 sec, without CCT
* Teacher's Explanation with EX&EK (#4,#5) is not right
* All the student responses are brief with one word. Not a sentence

3033DB-2007-04-12 p01 Ep07 w/o CCT [45:15 ~ 64:28] (many students' off topic talk)

Knowledge Reasoning / **Discourse Type**		Everyday Reasoning (EDR)		Knowledge		Scientific Reasoning (SR)			N/A
		Ss' Explanation (NE)	Ss' Experience (EX)	Ss' Knowledge (EK)	Scientific knowledge (SK)	Observation/ Data (OD)	**Patterns from Data (PD)**	Models/ Theories (MT)	
Explanation/Examples					2,2.5,2.6,2.12,2.13,2.14,2.15,2.16,2.17,2.18,2.19,2.20,2.22,2.23,2.24,2.25,8.1,8.2,8.3,8.4,8.5,8.6,8.7				1,1.1,1.2,1.3,1.4,1.5,1.6,1.7,1.8,1.9,1.10,1.11,1.12,2.1,2.2,2.3,2.4,2.7,2.8,2.9,2.10,2.11,2.28,3,3.6,3.9,4,4.1,4.2,4.3,4.5,5.3,5.5,5.6,5.6,5.17,5.18,5.19,7.1,7.2,7.3,7.4,9,9.1,9.2,9.3,9.4
Question/Prompt					2.21,2.26,3.7,3.8,4.4,4.6,5,5.1,6.2,7,7.9,8	3.1,3.3,3.4,3.10,6,6.1,			
Student Response/Question					2.21,4.6,5.1,6.2,7.6	3.1,3.3,3.4,3.5,3.10,3.11			2.26,2.27,5.19,7,7.3,7.8,7.9,8.7
Feedback/ prompt	L3:Elaborative								
	L2:Corrective				4.6,5.1,5.2,6.2,7.5,q7.6,7.6,7.9	3.1,q3.4,3.11			2.26,2.28,3.2,5.19,7
	L1:Evaluative								

* Time of the Episode: sec, Average Teachers' Response Time < 1 sec, without CCT
* Teacher's Explanation with EX&EK (#4,#5) is not right
* All the student responses are brief with one word. Not a sentence

3033DB-2007-04-12 P3 Ep01 CCT [17:13 ~ 21:11] the same content with previous one but different class

Knowledge / Reasoning — Discourse Type		Everyday Reasoning (EDR)		Knowledge		Scientific Reasoning (SR)			N/A
		Ss' Explanation (NE)	Ss' Experience (EX)	Naive Knowledge (EK)	Scientific knowledge (SK)	Observation/ Data (OD)	Patterns from Data (PD)	Models/ Theories (MT)	
Explanation/Examples					1,1.1,1.2,1.3,1.4,1.5,1.6,1.7,1.8,1.9,1.10,1.11,1.12,1.13,1.14,1.15,1.16,1.17,1.18,1.19,1.20				2.2,2.4,3,3.1,3.2,3.3,3.4,3.7,3.8
Question/Prompt			2,2.3						
Student Response/Question									2,2.1,2.3,2.4,2.5,3.4,3.5,3.8
Feedback/ prompt	L3:Elaborative								2.4,3.4,3.6,3.8
	L2:Corrective		2.1						
	L1:Evaluative								

* Topic: Electronic Power, Type: Lecture, Review Before Lab, CCT: No
* # of Dialogs:6, length of D(no EX):n/a R area: All SK
* # of Utterances:42, teacher's:24, students':0, N/A:21
* # of T's Utterances in: EX:3, SK:21, OD:0, PD:0, E:21, Q:2, Feedback: 11, L1:2, L2:9, L3:0
* # of Ss' Questions: 0

3033DB-2008-05-21P3 Ep01 w/o CCT [18:39 ~ 26:22] a lot of students' talk and the class was out of control

Discourse Type \ Knowledge Reasoning		Everyday Reasoning (EDR)		Knowledge		Scientific Reasoning (SR)			N/A
		Ss' Explanation (NE)	Ss' Experience (EX)	Naive Knowledge (EK)	Scientific knowledge (SK)	Observation/ Data (OD)	Patterns from Data (PD)	Models/ Theories (MT)	
Explanation/Examples					20,20.1,20.2,20.3,20.4,20.5,20.6,20.7,20.8,20.9,20.10,20.11				1,1.4,5.3,6.3,6.4,6.6,6.7,7.2,16.1,16.3,18.3,18.4
Question/Prompt					1,2,2.4,3,4,5,6,7,8,9,10,11,12,13,14,15,16,17,18,19				
Student Response/Question					1,2,2.3,3,4,5,6,6.2,6.4,6.7,6.10,7,7.2,8,9,9.1,9.2,10,10.1,10.2,11,12,13,14,15,16.2,17.1,18,19,20.3,20.9				1.1,1.3,2,2.1,2.2,3.1,3.2,5.1,5.2,6.1,6.5,6.9,12.1,16,16.1,16.2,17,18.2,
Feedback/ prompt	L3:Elaborative				q6.7,6.8				1.2,2.1,2.2,3.1,7,7.1,12.1,16.1,16.2,18.1
	L2:Corrective				q6.2,6.9,q6.10,6.10,q10.1,q10.2,18.4,20.9				
	L1:Evaluative				1.3,2.3,4,5.1,8,9,9.1,9.2,11,14,15,18,19,20.3				

* Topic: Current & Motor, Type: Lecture Before Lab, CCT: No
* # of Dialogs:20, length of D(no EX): 2,3,2,2,2,9,3,2,6,4,2,2,2,2,2,2,2,2,2,3, R area: All SK
* # of Utterances:127, teacher's:56, students':31, N/A:40
* # of T's Utterances in: EX:0, SK:87, OD:0, PD:0, E:12, Q:20, Feedback: 24, L1:14, L2:8, L3:2
* # of Ss' Questions: 0

3033DB-2008-05-22p1 Ep02 w CCT [19:53 ~ 25:40] watching the students' responses on the screen.

Discourse Type \ Knowledge Reasoning		Everyday Reasoning (EDR)		Knowledge		Scientific Reasoning (SR)			N/A
		Ss' Explanation (NE)	Ss' Experience (EX)	Naive Knowledge (EK)	Scientific knowledge (SK)	Observation/ Data/ (OD)	Patterns from Data (PD)	Models/ Theories (MT)	
Explanation/Examples					25,25.1,25.2,25.3,25.4,25.5,25.6,25.7,25.8,25.9,25.10,25.11,25.12				1,1.1,2,14.1,15,18,18.2,19,20,22,23,23.1,23.2,23.3,24
Question/Prompt					1				
Student Response/Question					cR,7.1,12				16,18,20.1,21,22,23.4
Feedback/ prompt	L3:Elaborative								8,16.1,18,18.1,19.1,21
	L2:Corrective				q12,12				
	L1:Evaluative				1.2,1.3,2,2.1,3,4,5,6,7,8.1,9,10,11,13,14,15.1,15.2,16,24.1,24.2				

* Topic: Speed & Frequency, Type: Lecture, CCT: Quick poll
* # of Dialogs:25, length of D(no EX):all less than2, R area: All SK (the teacher just read students' answers without any feedback –> L1)
* # of Utterances:66, teacher's:37, students':2 cR, N/A:27
* # of T's Utterances in: EX:0, SK:39, OD:0, PD:0, E:13, Q:1, Feedback: 24, L1:20, L2:2, L3:0
* # of Ss' Questions: 0

3033DB-2008-05-23p3 Ep01 w CCT [25:53 ~ 34:10] watching the students' responses on the screen.

Knowledge / Reasoning — **Discourse Type**		Everyday Reasoning (EDR) Ss' Explanation (NE)	Everyday Reasoning (EDR) Ss' Experience (EX)	Knowledge: Naive Knowledge (EK)	Knowledge: Scientific knowledge (SK)	Scientific Reasoning (SR) Observation/ Data/ (OD)	Scientific Reasoning (SR) Patterns from Data (PD)	Scientific Reasoning (SR) Models/ Theories (MT)	N/A
Explanation/Examples					22.2,26,26.1,26.2,26.3,26.4,26.5,26.6,26.7,26.8,26.9,26.10,26.11,26.12,26.13,26.14				1,1.1,1.2,2.1,2.2,8.1,20.1,20.2,20.3,20.5,20.7,21.1,21.2,21.4,21.5,
Question/Prompt					21				
Student Response/Question					cR,16				9,9.1,20.1,20.2,20.4,20.5,20.6,21,21.1,21.2,21.3,21.6,21.7,21.8,21.9,22.2
Feedback/ prompt	L3:Elaborative								9.1,20.5
	L2:Corrective				q16				
	L1:Evaluative				2,3,4,5,6,7,8,9,10,11,12,13,14,15,17,18,19,20,22,22.1,23,24,25,25.1,25.2				

* Topic: Speed & Frequency, Type: Lecture, CCT: Quick poll
* # of Dialogs:26, length of D(no EX): 1,2,2,2,2,2,2,2,2,2,2,2,2,2,2,2,3,2,2,2,2,2,2,2,2,1, R area: All SK (the teacher just read students' answers without any feedback –> L1)
* # of Utterances:77, teacher's:43, students':1 cR, N/A:33
* # of T's Utterances in: EX:0, SK:44, OD:0, PD:0, E:16, Q:1, Feedback: 26, L1:25, L2:1, L3:0
* # of Ss' Questions: 0

3050JM-2007-2-26 P1 Ep1.1 w/ CCT [3:45 ~ 7:10]

Knowledge Reasoning / **Discourse Type**		Everyday Reasoning (EDR)		Knowledge		Scientific Reasoning (SR)			N/A
		Ss' Explanation (NE)	Ss' Experience (EX)	Naive Knowledge (EK)	Scientific knowledge (SK)	Observation/ Data/ (OD)	Patterns from Data (PD)	Models/ Theories (MT)	
Explanation/Examples						1.1			1,1.4,1.5,1.6
Question/Prompt						1.2,1.3,			
Student Response/Question						cR,1.6,1.7,2,2.1,2.2,2.3,2.4,3,3.1,3.2,3.3,4,5,5.1,6,7			
Feedback/ prompt	L3:Elaborative					q2.1,q2.2,q2.3,q2.4,q3.3,q5.1,q7			
	L2:Corrective					q1.6,1.7,2,2.1,,2.3,2.4,q3,3.2,q4,q5,q6,6.1,7.1			
	L1:Evaluative								

* Topic: Speed, Type: Review PS with QP, CCT: QP
* # of Dialogs:7, **length of D**: 4,10,3,2,4,3,3 # of Reasoning Areas: all OD
* # of Utterances:44, teacher's:24, students':16, N/A:4
* # of T's Utterances in: EX:0, SK:0, OD:40, PD:0, E:1, Q:2, Feedback:21, L1:0, L2:14, L3:7
* # of Ss' Questions: 0

3050JM-2007-2-26 P1 Ep1.2 w/ CCT [7:15 ~ 10:41]

Knowledge Reasoning / **Discourse Type**		Everyday Reasoning (EDR)		Knowledge		Scientific Reasoning (SR)			N/A
		Ss' Explanation (NE)	Ss' Experience (EX)	Naive Knowledge (EK)	Scientific knowledge (SK)	Observation/ Data/ (OD)	Patterns from Data (PD)	Models/ Theories (MT)	
Explanation/Examples						1.4,1.8			1,1.1,1.3,1.6,1.7,1.11,1.12,1.13
Question/Prompt					3.2,3.4	1.5,1.9,1.10,5.1			
Student Response/Question					3.2,3.3,3.4	cR,2,3,3.1,3.5,4.1,4.2,5			
Feedback/ prompt	L3:Elaborative					q3.1,q3.3,q3.5,q4.2			
	L2:Corrective				3.4	q2,q3,3.5,4,q4.1,4.2,q5,5			
	L1:Evaluative								

* Topic: Speed, Type: Review PS with QP, CCT: QP
* # of Dialogs:5, **length of D**: 1,2,11,4,3 # of Reasoning Areas: all OD except 3(OD,SK)
* # of Utterances:39, teacher's:21, students':10, N/A:8
* # of T's Utterances in: EX:0, SK:6, OD:25, PD:0, E:2, Q:6, Feedback:13, L1:0, L2:9, L3:4
* # of Ss' Questions: 0

3050JM-2007-2-26 P1 Ep2 w/o CCT [11:39 ~ 18:05]

Knowledge / Reasoning — **Discourse Type**		Everyday Reasoning (EDR)		Knowledge		Scientific Reasoning (SR)			N/A
		Ss' Explanation (NE)	Ss' Experience (EX)	Naive Knowledge (EK)	Scientific knowledge (SK)	Observation/ Data/ (OD)	Patterns from Data (PD)	Models/ Theories (MT)	
Explanation/Examples					5.6,5.7,5.8	1,1.1,1.4,1.5,1.6,1.7,1.8,1.9,2,2.1,2.2,2.3,2.4,2.5,3,3.1,3.2,3.3,3.4,3.5,4,4.3,4.4,4.5,5.1,5.2,5.3,5.4,5.9,5.10,5.11,5.12,5.13,5.14,5.15,5.16,7,7.1,7.2,7.3,7.12,7.13,8,8.2,10,10.3,	1.10,4.1,4.2,5.5,7.10,7.11,10.4		
Question/Prompt						1.2,1.3,2.5,3.6,4.6,5,6,8.1,8.3,9	7.4,7.6,8.4,9.1,10.1		
Student Response/Question						1.2,1.3,2.6,3.6,3.7,4.6,5,6,8.1,8.3,9	6.1,6.2,7.4,7.5,7.7,7.8,7.9,7.10,8.4,8.5,8.6,9.1,9.2,9.3,10.1,10.2		
Feedback/ prompt	L3:Elaborative					q2.6,q3.7,	q6.1,6.3,q7.5,q7.7,q7.8,q7.9,q7.10,q8.5,q10.2		
	L2:Corrective					1.2,2.6,4.6,5,6	6.3,q7.6,7.9,9.2,q9.3,10.2		
	L1:Evaluative								

* Topic: Speed, distance graph, average speed, **Complex Motion**, Type: Lecture, CCT: no
* # of Dialogs:10, **length of D**: 5,3,3,2,2,4,11,7,5,4 # of Reasoning Areas: 1,2,3,4(OD), 5(OD,SK,PD), 6(OD,PD), 7,8,9,10(OD,PD),
* # of Utterances:120, teacher's:93, students':27, N/A:0
* # of T's Utterances in: EX:0, SK:3, OD:74, PD:43, E:56, Q:15, Feedback:22, L1:0, L2:11, L3:11
* # of Ss' Questions: 0

3050JM-2007-2-26 P1 Ep3.1 w CCT-EX [19:32 ~ 25:28]

Knowledge Reasoning / **Discourse Type**		Everyday Reasoning (EDR)		Knowledge		Scientific Reasoning (SR)			N/A
		Ss' Explanation (NE)	Ss' Experience (EX)	Naive Knowledge (EK)	Scientific knowledge (SK)	Observation/ Data/ (OD)	Patterns from Data (PD)	Models/ Theories (MT)	
Explanation/Examples						1,1.1,1.2,3.3,3.4,3.5	3.2,		
Question/Prompt						1,1.1,1.2,1.3,1.4,2	3,3.1,3.6,3.7,		
Student Response/Question						1.4,1.5,2	2.1,2.2,2.3,2.4,3.1,3.7,3.8,3.9,3.10		
Feedback/ prompt	L3:Elaborative						q2.1,q2.2,q3.8		
	L2:Corrective					1.4,1.5	2.4,3.1,q3.10,3.10		
	L1:Evaluative								

* Topic: Complex Motion, Graph, Type: Review after demonstration, CCT: AC graph
* # of Dialogs:3, **length of D**: 4,6,8 # of Reasoning Areas: OD, (OD,PD), OD,PD)
* # of Utterances:38, teacher's:26, students':12, N/A:0
* # of T's Utterances in: EX:0, SK:0, OD:17, PD:21, E:7, Q:10, Feedback:9, L1:0, L2:6, L3:3
* # of Ss' Questions: 0

3050JM-2007-2-26 P1 Ep3.2 w CCT-EX [27:58 ~ 27:34]

Knowledge Reasoning / **Discourse Type**		Everyday Reasoning (EDR)		Knowledge		Scientific Reasoning (SR)			N/A
		Ss' Explanation (NE)	Ss' Experience (EX)	Naive Knowledge (EK)	Scientific knowledge (SK)	Observation/ Data/ (OD)	Patterns from Data (PD)	Models/ Theories (MT)	
Explanation/Examples						1.1,1.2,1.3	1.4,1.5		1,3
Question/Prompt							1.6,2,		
Student Response/Question						1.6,1.7,3.1	2,2.1,2.2,q2.2,2.3,3.2		
Feedback/ prompt	L3:Elaborative					q3.1	q2.2,3.2		
	L2:Corrective					1.6,	1.7,2.1,q2.3, 2.3,3.2		
	L1:Evaluative								

* Topic: Complex Motion Graph, Type: Review after demonstration, CCT: AC graph
* # of Dialogs:3, length of D(no EX):4,7,5 # of Reasoning Areas: all (OD,PD)
* # of Utterances:26, teacher's:17, students':9, N/A:1
* # of T's Utterances in: EX:0, SK:0, OD:8, PD:17, E:5, Q:2, Feedback:9, L1:0, L2:6, L3:3
* # of Ss' Questions: 0

3050JM-2007-2-26 P1 Ep3.3 w CCT-EX [28:40 ~ 29:44]

Knowledge Reasoning / **Discourse Type**		Everyday Reasoning (EDR)		Knowledge		Scientific Reasoning (SR)			N/A
		Ss' Explanation (NE)	Ss' Experience (EX)	Naive Knowledge (EK)	Scientific knowledge (SK)	Observation/ Data/ (OD)	Patterns from Data (PD)	Models/ Theories (MT)	
Explanation/Examples						1,1.1,3.2,3.3,			
Question/Prompt						2,3,4			
Student Response/Question						1.2,1.3,1.4,2,2 .1,3,3.1,4,4.1			
Feedback/ prompt	L3:Elaborative					q1.3,q1.4,q4.1			
	L2:Corrective					1.2,1.4,2.1,3.1 ,4,4.1(girl)			
	L1:Evaluative								

* Topic: Complex Motion Graph, Type: Review after demonstration, CCT: AC graph
* # of Dialogs:4, **length of D**: 6,2,2,4, # of Reasoning Areas: all OD
* # of Utterances:25, teacher's:16, students':9, N/A:0
* # of T's Utterances in: EX:0, SK:0, OD:25, PD:0, E:4, Q:3, Feedback:9, L1:0, L2:6, L3:3
* # of Ss' Questions: 0

3050JM-2007-02-26P3 Ep1.1 w CCT [2:41 ~ 2:50, 5:30 ~ 6:49] 3minutes waiting after Q1, compare with 3033 in SK

Knowledge Reasoning / **Discourse Type**		Everyday Reasoning (EDR)		Knowledge		Scientific Reasoning (SR)			N/A
		Ss' Explanation (NE)	Ss' Experience (EX)	Naive Knowledge (EK)	Scientific knowledge (SK)	Observation/ Data (OD)	Patterns from Data (PD)	Models/ Theories (MT)	
Explanation/Examples									1.1,1.3
Question/Prompt					1,1.2,2.1,2.2,2.3				
Student Response/Question					cR,1.4,2,2.1,2.2,2.3,3,4,4.1,5,6,7,7.1,8,8.1,8.2				
Feedback/ prompt	L3:Elaborative				q1.4,q2,2.3,q3,3.1,q4,q5,q6,q7,q7.1,q8,q8.1,q8.2				
	L2:Corrective				1.4,2.2,3,6,7.1,8.2				
	L1:Evaluative								

* Topic: Formula of Speed Type: Review PS with QP, CCT: QP
* # of Dialogs:8, **length of D**: 4,9,3,2,2,3,5,7 # of Reasoning Areas: all SK
* # of Utterances:41, teacher's:24, students':15, N/A:2
* # of T's Utterances in: EX:0, SK:41, OD:0, PD:0, E:0, Q:5, Feedback:19, L1:0, L2:6, L3:13
* # of Ss' Questions: 0

3050JM-2007-02-26P3 Ep1.2 w/ CCT [7:09~7:15, 9:20 ~ 10:47] 2 minutes waiting after Q1

Knowledge / Reasoning — **Discourse Type**		Everyday Reasoning (EDR)		Knowledge		Scientific Reasoning (SR)			N/A
		Ss' Explanation (NE)	Ss' Experience (EX)	Naive Knowledge (EK)	Scientific knowledge (SK)	Observation/ Data (OD)	Patterns from Data (PD)	Models/ Theories (MT)	
Explanation/Examples						1,1.2,4.1,8.3			1.3,1.4,1.5, 1.6
Question/Prompt					2,2.1,4.2	1.1,3,4,8.1			
Student Response/Question					2.1,2.2,4.2	cR,3,4,5,6.1,7 ,8,8.1,8.2			1.6,1.7
Feedback/ prompt	L3:Elaborative					q5,6.1,q7,q8			
	L2:Corrective				2.2,4.2	6,6.1,7,8.1,8.2			
	L1:Evaluative								

* Topic: Calculating Average Speed Type: Review PS with QP, CCT: QP
* # of Dialogs:8, **length of D**: 2,3,3,5,2,3,3,7 # of Reasoning Areas: all OD but 2(OD,SK), 4(OD,SK)
* # of Utterances:39, teacher's:22, students':11, N/A:6
* # of T's Utterances in: EX:0, SK:8, OD:25, PD:0, E:4, Q:6, Feedback:11, L1:0, L2:7, L3:4
* # of Ss' Questions: 0

3050JM-2007-02-26P3 Ep02 w/o CCT [11:25 ~ 17:42] (compare to 3033 lecture)

Knowledge Reasoning / **Discourse Type**		Everyday Reasoning (EDR)		Knowledge		Scientific Reasoning (SR)			N/A
		Ss' Explanation (NE)	Ss' Experience (EX)	Naive Knowledge (NK)	Scientific knowledge (SK)	Observation/ Data (OD)	Patterns from Data (PD)	Models/ Theories (MT)	
Explanation/Examples						1,1.1,1.2,1.3, 1.4,1.5,1.6,1.7 ,1.8,2,2.1,2.2, 2.4,2.5,2.6,2.7 ,3.3,3.4,3.5,3. 6,3.7,3.8,4,4.1 ,4.2,4.3,5,5.1, 5.2,5.3,5.4,5.5 ,5.6,5.7,5.8,5. 9,5.10,6,6.1,6. 2,6.3,8.5,9,	6.7,6.8,6.9,7. 5,8.6,8.9,9.3, 9.4,9.5,10.6, 10.7,10.8,10. 9,10.10,10.1 1		3.2,6.6,10,1 0.4,10.5
Question/Prompt					3,3.1	2.3,3.9,4.4,5.1 1,8.3,8.4	6.4,6.5,7,8,8. 7,9.1,9.2,10. 1		
Student Response/Question					3,3.1	2.3,4.4,5.11,8. 4	6.4,7,7.1,7.2, 7.3,7.4,8,8.1, 8.2,8.7,9.1,9. 2,10.1,10.2,1 0.3		
Feedback/ prompt	L3:Elaborative						q7.2,7.3,q7.4 ,8.7		
	L2:Corrective				3	2.3,3.9,4.4,8.4	q8.2,9.1,9.2		
	L1:Evaluative								

* Topic: Complex Motion Type: Lecture, CCT: No
* # of Dialogs:10, **length of D**: 0,2,4,2,1,2,6,8,4,2 # of Reasoning Areas: all OD but 3(SK,OD), 6,7,8,9(OD,PD)
* # of Utterances:114, teacher's:88, students':21, N/A:5
* # of T's Utterances in: EX:0, SK:5, OD:67, PD:37, E:60, Q:16, Feedback:12, L1:0, L2:8, L3:4
* # of Ss' Questions: 0

3050DB-2007-02-27p1 Ep01 w CCT [16:03 ~ 20:56] Review class (should be compared to 3033)

Knowledge Reasoning / **Discourse Type**		Everyday Reasoning (EDR)		Knowledge		Scientific Reasoning (SR)			N/A
		Ss' Explanation (NE)	Ss' Experience (EX)	Naive Knowledge **(NK)**	Scientific knowledge (SK)	Observation/ Data/ (OD)	Patterns from Data (PD)	Models/ Theories (MT)	
Explanation/Examples			1.13,1.14,1.15 ,1.16,1.17	3.1,3.2,3.3, 3.5	1,1.2,1.5,5. 5,	1.1,1.6,1.7,1.8 ,1.9,1.10,1.11, 4,	1.3,1.4,2.5,5, 5.1,		1.18,1.19,1. 20
Question/Prompt				3.4	1.12,2,5.6	4.1,4.2	2.2,3,3.6,3.7, 5.2,5.3,5.4		
Student Response/Question				3.4	cR,1.12,2,2 .1,5.6	4.1,4.2,4.3	2.2,2.3,3,3.6, 3.7,3.8,5.4		
Feedback/ prompt	L3:Elaborative				2,2.1	q4.3	q2.3,q3.8		
	L2:Corrective			3.4	1.12,5.6	4.1,4.3	2.3,2.4,3,3.6, 3.8,5.4,		
	L1:Evaluative								

* Topic: Complex Motion, Position Graph, Type: Review PS after PS with LC, CCT: LC
* # of Dialogs:5, **length of D**: 3,9,10,6,4, # of Reasoning Areas: (SK,EX,OD,PD),(SK,PD),(PD,NK), (OD),(PD,SK)
* # of Utterances:73, teacher's:54, students':15, N/A:4
* # of T's Utterances in: EX:5,NK:7, SK:15, OD:16, PD:27, E:26, Q:13, Feedback:16, L1:0, L2:11, L3:5
* # of Ss' Questions: 0

3050DB-2007-02-27p1 Ep2.1 w CCT [38:38 ~ 51:53] Review class (should be compared to 3033)

Knowledge Reasoning / **Discourse Type**		Everyday Reasoning (EDR)		Knowledge		Scientific Reasoning (SR)			N/A
		Ss' Explanation (NE)	Ss' Experience (EX)	Naive Knowledge (EK)	Scientific knowledge (SK)	Observation/ Data/ (OD)	Patterns from Data (PD)	Models/ Theories (MT)	
Explanation/Examples						1.1,2.1,2.2,2.5	4.5		1,1.2,1.3,1.4,1.5,1.6,2.7,2.9,
Question/Prompt						2,2.1,2.3,2.8,2.10,3.3	4,4.1,4.6,5,5.2,6		
Student Response/Question						q2.4,2.5,2.6,cR,2.12,2.14,2.15,q2.16,2.19,2.20,2.21,3.2,3.3,3.4,3.5	4.1,4.2,4.3,4.4,4.6,5,5.1,5.2,6		1.6,1.7,2.3,3,3.1
Feedback/ prompt	L3:Elaborative					q2.18,q3,q3.2	q4.6,q5.1		2.3,2.11,2.13,2.17,3.1
	L2:Corrective					2.4,2.14,3.5	4.4,5.1,6.1		
	L1:Evaluative								

* Topic: Complex Motion, Position Graph, Type: Review after Lab, CCT: QP
* # of Dialogs:6, length of D(no EX):0,9,4,6,5,2 # of Reasoning Areas: all OD,PD
* # of Utterances:70, teacher's:28, students':24, N/A:18
* # of T's Utterances in: EX:0,NK:0, SK:0, OD:31, PD:21, E:5, Q:12, Feedback:11, L1:0, L2:6, L3:5
* # of Ss' Questions: 0

3050DB-2007-02-27p1 Ep2.2 w CCT [38:38 ~ 51:53] Review class (should be compared to 3033)

Knowledge / Reasoning / **Discourse Type**		Everyday Reasoning (EDR) Ss' Explanation (NE)	Everyday Reasoning (EDR) Ss' Experience (EX)	Knowledge: Naive Knowledge (EK)	Knowledge: Scientific knowledge (SK)	Scientific Reasoning (SR) Observation/ Data/ (OD)	Scientific Reasoning (SR) Patterns from Data (PD)	Scientific Reasoning (SR) Models/ Theories (MT)	N/A
Explanation/Examples						7.3,7.4,8.1,8.2 ,			7,7.6
Question/Prompt						7.1,7.2,7.7,8,9			
Student Response/Question						7.4,7.6,cR,7.9 ,7.10,7.11,7.1 2,8,9,9.1,9.2,			7.5
Feedback/ prompt	L3:Elaborative					q7.11,q9.2			7.5,7.8
	L2:Corrective					q7.10,7.12,8,9 .1			
	L1:Evaluative								

* Topic: Complex Motion, Position Graph, Type: Review after Lab, CCT: QP
* # of Dialogs:3, length of D(no EX):7,2,3, , # of Reasoning Areas: all OD
* # of Utterances:31, teacher's:15, students':11, N/A:5
* # of T's Utterances in: EX:0,NK:0, SK:0, OD:26, PD:0, E:4, Q:5, Feedback:6, L1:0, L2:4, L3:2
* # of Ss' Questions: 0

3050DB-2007-02-27p1 Ep2.3 w CCT [38:38 ~ 51:53] Review class (should be compared to 3033)

Knowledge Reasoning / **Discourse Type**		Everyday Reasoning (EDR)		Knowledge		Scientific Reasoning (SR)			N/A
		Ss' Explanation (NE)	Ss' Experience (EX)	Naive Knowledge (EK)	Scientific knowledge (SK)	Observation/ Data/ (OD)	Patterns from Data (PD)	Models/ Theories (MT)	
Explanation/Examples						10.1,10.9,10.16,12.4			10,10.2,10.4,10.5,10.11,10.12,10.14,10.18
Question/Prompt						10.3,10.6,10.7,10.8,			
Student Response/Question						cR,10.19,12,	12.1,12.2,12.3		10.7,10.12,10.15,10.18
Feedback/ prompt	L3:Elaborative						q12.1,q12.2,q12.3		10.7,10.13,10.16,10.17,10.18
	L2:Corrective					10.15,q12,12.1,12.2,12.5	12.3		
	L1:Evaluative								

* Topic: Complex Motion, Position Graph, Type: Review after Lab, CCT: QP
* # of Dialogs:2, length of D(no EX): 3,8, # of Reasoning Areas: (OD), (OD,PD)
* # of Utterances:39, teacher's:17, students':5, N/A:17
* # of T's Utterances in: EX:0,NK:0, SK:0, OD:15, PD:7, E:4, Q:4, Feedback:9, L1:0, L2:6, L3:3
* # of Ss' Questions: 0

3050DB-2007-02-27p3 Ep01 w CCT [10:30 ~ 17:56] Review class (should be compared to 3033)

Knowledge Reasoning / **Discourse Type**		Everyday Reasoning (EDR)		Knowledge		Scientific Reasoning (SR)			N/A
		Ss' Explanation (NE)	Ss' Experience (EX)	Naive Knowledge (NK)	Scientific knowledge (SK)	Observation/ Data/ (OD)	Patterns from Data (PD)	Models/ Theories (MT)	
Explanation/Examples			3.7,3.9	4.3,4.6	1,9,9.1	3,3.2,3.3,3.4,3.5,3.6,3.10,3.11,3.12,4.7,4.8,5,5.5,6,6.1,9.3,9.4,9.5,10.7,11,	3.7,3.8,4.1,4.2,4.4,4.5,5.6,9.6,10.2,10.8,10.9,11.1,11.2,11.5,11.6		10.3,10.4,10.5
Question/Prompt					1,2	4.8,5.1,7,7.1	2.1,2.2,4,8,10,11.3,11.4		
Student Response/Question					cR,1,1.1,2	3.1,4.8,5.1,5.2,7.1	2.2,4,8,10,10.1,11.4		10.5
Feedback/ prompt	L3:Elaborative								
	L2:Corrective				1,2	3.1,4.8,5.2,q5.3,5.4,7.1,11.9,11.10,11.12	10.1,		10.6,11.7,11.8,11.11
	L1:Evaluative								

* Topic: Complex Motion, Position Graph, Type: Review after Lab, CCT: LC
* # of Dialogs:11, **length of D**: 3,4,2,4,5,0,3,2,0,3,5, # of Reasoning Areas: all (OD,PD) but 3(OD,PD,EX),4(OD,PD,NK),9(OD,PD,SK)
* # of Utterances:89, teacher's:67, students':14, N/A:8
* # of T's Utterances in: EX:2,NK:2, SK:10, OD:38, PD:29, E:42, Q:13, Feedback:12, L1:0, L2:12, L3:0
* # of Ss' Questions: 0

Example) T: And then when you walked towards the CBR, Adam, the graph changed again. E1.4 Each segment, or smaller event of your travels, we call a leg, ok? E1.5 And that's why we call it complex motion; it's when we do things… E1.6 We go on vacation. You don't drive from Canton to Myrtle Beach at 60 MPH without ever stopping. E1.7 It's not one linear straight line graph, is it? You stop at McDonald's, you stop for gas, sometimes you get tied up in traffic and you have to drive slower,E1.8 We can break that trip down into different legs of motion: E1.9 the times when we move slower, the times when we are stopped, the times when we move faster. E1.10 Then, we have to turn around and come home, so our position would look different. E1.11 It would go downward. E1.12 Ok?

3050DB-2007-02-27p3 Ep02 w CCT [34:05 ~ 41:20] Review class (should be compared to 3033)

Knowledge Reasoning / **Discourse Type**		Everyday Reasoning (EDR)		Knowledge		Scientific Reasoning (SR)			N/A
		Ss' Explanation (NE)	Ss' Experience (EX)	Naive Knowledge (EK)	Scientific knowledge (SK)	Observation/ Data/ (OD)	Patterns from Data (PD)	Models/ Theories (MT)	
Explanation/Examples						1.6,2.1,2.2,2.3	3.5,3.6,3.10		1,1.1,1.2,1.4,1.9,1.12,1.13,1.14,1.15,3,3.1,3.4,3.7,3.8,3.9,5.3,5.4,5.6,5.9,5.10,5.11
Question/Prompt						1.3,1.5,1.7,1.8,1.10,1.11,6	3.2,3.3,4,4.1,5,5.1,5.2,5.5		
Student Response/Question						cR,1.16,1.17,1.18,1.19,2,2.3,cR,6.1,6.2,6.3,6.4	3.9,3.10,4,4.1,4.2,5.3,5.5,5.6,5.7,5.8,5.13,5.15		1.10,5.4
Feedback/ prompt	L3:Elaborative					q2,q6.2			1.10,3.11,5.12
	L2:Corrective					q1.16,2,2.3,q6.4,6.4	q3.10,3.10,4.1,4.2,5.13,5.14,5.15		
	L1:Evaluative								

* Topic: Complex Motion, Position Graph, Type: Review after Lab, CCT: QP
* # of Dialogs:6, **length of D:** 4,3,4,5,7,5, # of Reasoning Areas: all (OD,PD)
* # of Utterances:76, teacher's:36, students':14, N/A:26
* # of T's Utterances in: EX:0,NK:0, SK:0, OD:28, PD:30, E:7, Q:15, Feedback:14, L1:0, L2:12, L3:2
* # of Ss' Questions: 0
